# Adaptive Effects of Endocrine Hormones on Metabolism of Macronutrients during Fasting and Starvation: A Scoping Review

**DOI:** 10.3390/metabo14060336

**Published:** 2024-06-16

**Authors:** Reza Karimi, Alina Yanovich, Fawzy Elbarbry, Anita Cleven

**Affiliations:** Pacific University School of Pharmacy, 222 SE 8th Avenue, HPC-Ste 451, Hillsboro, OR 97123, USA; alina.yanovich@pacificu.edu (A.Y.); fawzy.elbarbry@pacificu.edu (F.E.); anita.cleven@pacificu.edu (A.C.)

**Keywords:** fasting, starvation, metabolism, macronutrients, cortisol, insulin, glucagon, thyroid hormones

## Abstract

Food deprivation can occur for different reasons. Fasting (<24 h duration) occurs to meet religious or well-being goals. Starvation (>1-day duration) occurs when there is intentional (hunger strike or treatment of a medical condition) or unintentional (anorexia nervosa, drought, epidemic famine, war, or natural disaster) food deprivation. A scoping review was undertaken using the PubMed database to explore 1805 abstracts and review 88 eligible full-text articles to explore the adaptive relationships that emerge between cortisol, insulin, glucagon, and thyroid hormones on the metabolic pathways of macronutrients in humans during fasting and starvation. The collected data indicate that fasting and starvation prime the human body to increase cortisol levels and decrease the insulin/glucagon ratio and triiodothyronine (T_3_) levels. During fasting, increased levels of cortisol and a decreased insulin/glucagon ratio enhance glycogenolysis and reduce the peripheral uptake of glucose and glycogenesis, whereas decreased T_3_ levels potentially reduce glycogenolysis. During starvation, increased levels of cortisol and a decreased insulin/glucagon ratio enhance lipolysis, proteolysis, fatty acid and amino acid oxidation, ketogenesis, and ureagenesis, and decreased T_3_ levels reduce thermogenesis. We present a potential crosstalk between T_3_ and the above hormones, including between T_3_ and leptin, to extend their adaptive roles in the metabolism of endogenous macronutrients during food deprivation.

## 1. Introduction

The central nervous system (CNS) plays a vital role in the regulation of endocrine glands and their hormonal release to maintain energy homeostasis. An interruption in this physiologic role can lead to obesity and metabolic disorders [1]. Both fasting and starvation can affect the brain due to glucose deprivation, resulting in the synthesis and release of cortisol, epinephrine, and growth hormones to promote glucose homeostasis [2]. The effect of fasting and starvation on the CNS followed by the impact on different endocrine glands is inevitable [2]. For instance, the pancreas and its alpha and beta cells are affected during fasting and starvation, and the hormonal release from both types of cells and their effects on the hepatic and extrahepatic tissues are predictable [3]. Good examples of daily fasting are fasting that occurs during the month of Ramadan observed by the Islamic population, when healthy individuals experience abstention from food and drink of any kind including water from dawn to dusk, which can range from 12–22 h [3], or intermittent fasting (IF), which refers to a cycle of eating patterns of little to no caloric intake on a recurring basis that can range from 12 h to several days [3,4].

Anorexia nervosa (AN) is the most ethical representation of starvation. Anorexia nervosa is an eating and psychiatric disorder in which approximately half of the deaths are caused by physical and metabolic complications (malabsorption syndrome, diabetes mellitus, Addison’s disease, thyroid dysfunction, etc.) associated with starvation [5]. This condition is caused by severe and persistent food restriction [6] that leads to serious biochemical and physiological comorbidities and endocrine dysfunctions [7] and is associated with high mortality [8]. Nearly all organs and biochemical processes, including metabolic and electrolyte homeostasis, are affected by starvation as a result of insufficient access to essential molecules. While AN most commonly affects young women, it has been presented in adolescent boys and men as well [9]. Abnormalities in carbohydrate metabolism as a result of AN have been known since 1937, when Sheldon published an article discussing the metabolic consequences of AN [10]. To compensate for these consequences, the human body begins to inherently correlate with endocrine tissues and organs to release a series of hormones, significantly affecting the metabolism of endogenous macronutrients (carbohydrates, lipids, proteins) during fasting and starvation, particularly in the beginning stages [2,3].

As fasting and starvation differ in the duration of food deprivation, it is expected that endocrine hormones execute their stimulatory or inhibitory mechanisms to different extents. It has been reported that stress, fasting, and starvation cause high levels of cortisol release [11,12]. The pancreas is the fastest organ to respond to food deprivation by increasing serum levels of glucagon [13,14,15]. Comparatively, fasting and starvation reduce serum levels of insulin [16,17,18,19], triiodothyronine (T_3_) [20,21,22,23,24,25,26,27,28], and leptin [29,30].

While different endocrine hormones are affected differently during fasting and starvation, there is a growing body of evidence that when one endocrine gland is affected, other glands are also affected, either directly or indirectly. For instance, an increased level of cortisol can lead to insulin resistance and muscle atrophy [31]. Similarly, cortisol acts as an endogenous antagonist of leptin, and lower levels of leptin may contribute to increased cortisol levels during fasting [32]. Additionally, a reduced level of leptin results in reduced T_3_ and thyroid-stimulating hormone (TSH) levels [33,34], which is also associated with low levels of insulin and increased levels of cortisol [34]. All three sources of macronutrients—glycogen to release glucose, essential and expandable proteins to release amino acids, and triacylglycerols to release fatty acids and glycerol—play a critical role in fasting and the duration of survival in starvation [2,3].

We have been intrigued by the adaptive roles that endocrine hormones play in metabolism during both fasting and starvation. Endocrine hormones such as cortisol, glucagon, insulin, and T_3_ affect glucose homeostasis by influencing multiple organs and organ systems such as the liver, gastrointestinal tract, pancreas, adipose tissue, skeletal muscles, and the central nervous system. These hormones metabolize endogenous macronutrients to different extents during fasting and starvation [2,3,11,13,16,28,35]. While these studies have enriched our understanding of how one or more endocrine hormones affect the metabolism of endogenous macronutrients during fasting or starvation, there is no clear explanation for why different hormones produce the same metabolic outcomes within or between the above two food deprivations. In addition, it is not fully understood how these endocrine hormones apply their inhibitory or stimulatory effects to support each other during food deprivation. A parallel review of fasting and starvation that explores why the above endocrine hormones produce similar metabolic outcomes and how they support each other via their inhibitory or stimulatory actions to promote metabolic homeostasis has not been explored extensively. Therefore, the objective of this review study was to explore the available published data on these two nutrition deprivations and to summarize the adaptive and supportive crosstalk roles that the adrenal glands, the pancreas, the thyroid gland, and their hormones play in the major metabolic pathways of endogenous macronutrients. This objective was guided by the following research question: what are the adaptive stimulatory and inhibitory effects that cortisol, glucagon, insulin, and T_3_ hormones exert to support and/or promote metabolic homeostasis during fasting and starvation? The intention of the results and discussions presented herein is to spark curiosity in the scientific community to further research the impacts of endocrine organs on the metabolism of endogenous macronutrients during different forms of food deprivation.

## 2. Methods

This review is focused on human fasting and starvation. For the purposes of this review, fasting refers to food deprivation for less than 24 h in healthy individuals. Anorexia nervosa is a human model of chronic starvation and it has been considered as a representation of starvation [36]. Individuals with AN experience food deprivation that extends beyond 24 h. Some major and general similarities and differences between fasting and starvation are presented in Table 1, which will be expanded upon and discussed further in the results and discussions sections.

The scoping review approach was undertaken based on the Joanna Briggs Institute (JBI) methodology [40,41,42], and we searched available evidence to address the above objective and to highlight a few novel areas for further research studies. Our search began on 26 September 2023. The Preferred Reporting Items for Systematic Reviews and Meta-Analyses (PRISMA) for scoping reviews were used to direct our search strategy and assist us in presenting the results and outcomes of our review study [40]. The following subsections indicate details of the search strategies, selection process, data extraction, and synthesis.

### 2.1. Search Terms

The PubMed database was searched for eligible studies. The following search strings include the key terms used in the titles of abstracts of relevant published articles. Fasting search terms in the PubMed database were as follows: human fasting AND cortisol AND metabolic homeostasis; human fasting AND glucagon AND metabolic homeostasis; human fasting AND insulin release AND metabolic homeostasis; human fasting AND thyroid hormones AND metabolic homeostasis. Starvation search terms in the PubMed database were as follows: anorexia nervosa AND cortisol AND metabolism; anorexia nervosa AND glucagon AND metabolism; anorexia nervosa AND insulin AND metabolism; anorexia nervosa AND thyroid hormones AND metabolism. The time frame of publication for the abstracts and articles to be reviewed was set to be from 1971 to December 2023. We did not expect to find any starvation studies in humans due to ethical reasons. Much of our understanding of the roles that starvation plays on metabolism and adaptive neuroendocrine responses comes from studying patients with AN, and since 1977, there have been many studies that have included patients with AN as a model of starvation. As a result, we have used AN studies as the closest representation of starvation in humans.

Since fasting blood glucose measurements and the role of insulin have been extensively studied in diabetes, many articles have been produced on that subject (nearly 7000 articles). As a result, the terms for insulin search were slightly changed to “Human fasting AND insulin release AND metabolic homeostasis” in order to focus on the roles of insulin during fasting in healthy individuals.

### 2.2. Search Strategy

We conducted a literature search and focused on published peer-reviewed studies. While the majority of eligible full-text articles were reviews, we included clinical, interventional, and observational research that matched our inclusion criteria as well. In order to not overlook any pertinent data, each title and abstract were screened by at least two screeners (two authors in this review) to assess their suitability and ensure that the abstract matched the inclusion criteria presented in the following subsection. We removed unrelated publications. While the search produced a publication from a book chapter, the search did not include gray literature.

### 2.3. Inclusion Criteria

The search engine used was PubMed (text availability: abstract and full text), and three search elements—population, intervention, and outcome measures [41]—were applied to guide the search terms and assist us in answering the study’s objective and question. For instance, to indicate the roles of cortisol in the metabolism of macronutrients, the following terms were used: “human fasting” (or “anorexia nervosa”) to represent population; “cortisol” to represent intervention; and “metabolic homeostasis” to represent outcome measures. In order to narrow the focus of the searches to intervention (hormones that are uniquely associated with adaptive metabolism during food deprivation), the fourth search element, comparison intervention, was not used. Articles with a full text and abstract dating from 1971 to December 2023 that linked fasting and starvation to metabolic roles of cortisol, glucagon, insulin, and thyroid hormones were included. When a full article was not accessible but the abstract indicated data relevant to this review, the university library system was used to order the desired articles.

### 2.4. Exclusion Criteria

Pathological changes and medications may lead to disturbances in endocrine organs and the release of their hormones. As a result, articles related to the treatment of diseases, including chronic conditions, the use of medication therapies, or the impacts of unrelated hormones and other molecules, were excluded from the review. Based on the exclusion criteria, we identified 57 full-text articles that were not relevant to our review study.

### 2.5. Selection Process

All four authors of this article participated in the review of abstracts provided in the search to ensure that each published article matched the above inclusion criteria and that the article was relevant to this review. In case of any uncertainties regarding a link between the search words/parameters and the abstract, the full article was retrieved. The PRISMA flow diagram guidelines were followed [42], and an extraction spreadsheet was used to ensure that all necessary data were presented in this scoping review article [40].

### 2.6. Data Extraction and Synthesis

A structured extraction spreadsheet that included characteristics relevant to the review study was generated. The spreadsheet included information for authors, title of the study, journal name, year, aims/purpose, population and sample size, source of evidence, methodology, and outcomes of the published articles. We used a color code approach to indicate that at least two independent authors agreed that the identified results from abstracts matched the inclusion criteria. While we did not use any statistical software analysis to extract data, the identified data from published literature were manually extracted based on the above characteristics. After the search and extraction of data were complete, duplications and irrelevant extracted data were removed from the spreadsheet. The corresponding author evaluated the suitability of all relevant full-text articles, including reviewing the reference list of each included article, to identify information related to this study. Results were synthesized in a manner that matched the study characteristics, such as the type of publications, the roles of endocrine hormones, and the stimulatory and inhibitory metabolic impacts of endocrine hormones on endogenous macronutrients during different food deprivations in humans. Synthesized results were reviewed and discussed among the four authors to ensure that the results accurately reflected the objective and study question. Based on the identified results, data were summarized in two tables and one figure. We did not assess the risk of bias; however, to prevent any bias, contradicting results that were identified during synthesis are presented in the results section of this review study. For instance, while a vast number of published results indicated that glucagon levels increased during starvation, a few studies indicated that glucagon levels were reduced during starvation. Accordingly, we presented this contradicting finding in our results and referenced the published article(s).

## 3. Results

### 3.1. Characteristics of Included Work

**Figure 1:**
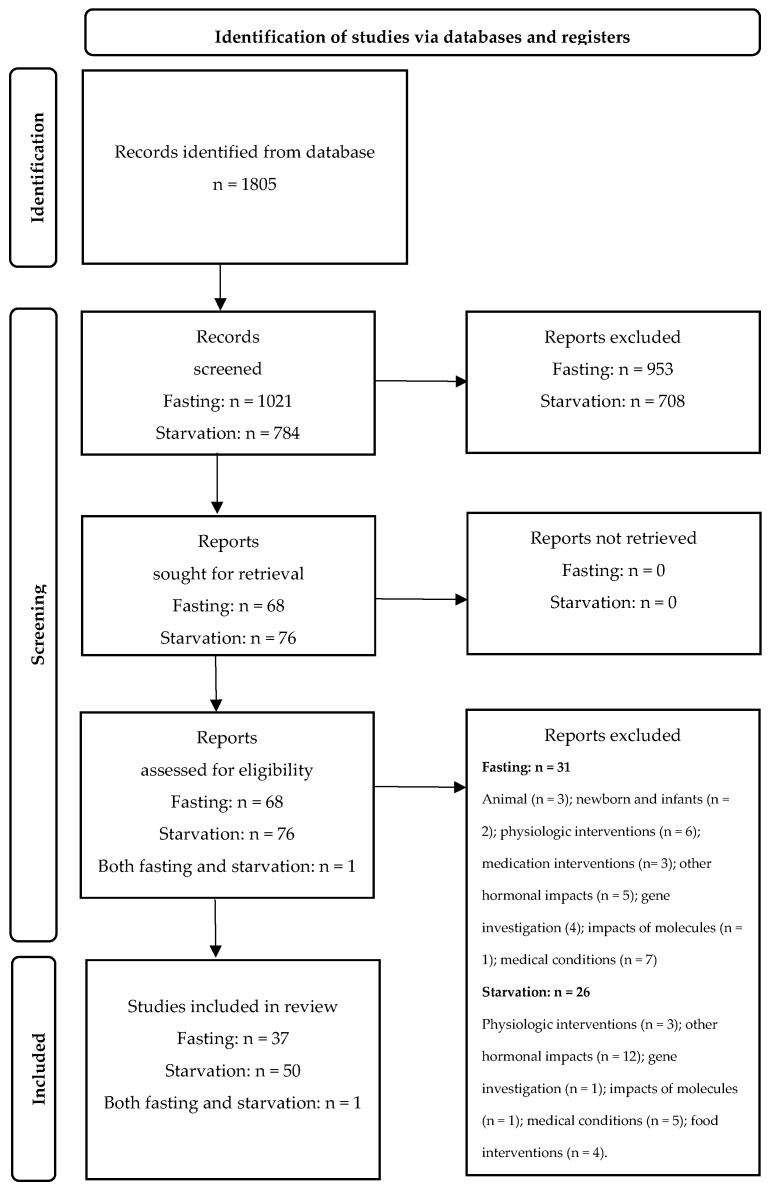
Preferred Reporting Items for Systematic Reviews and Meta-Analyses (PRISMA) flow diagram used to indicate the number of reports that were searched and identified from the PubMed database for the scoping review process [42].

In order to gain a better understanding of the impacts that cortisol, glucagon, insulin, and thyroid hormones have on the metabolism of endogenous macronutrients, we focused on two food deprivations, fasting and starvation. It is clear that one cannot draw conclusions about fasting based on starvation data and vice versa. As a result, a parallel search strategy was implemented to produce results from the above two food deprivations. A total of 1805 records were identified in the initial search for both fasting and starvation, and we immediately began reviewing abstracts. Upon reviewing the abstracts, a total of 1661 of the abstracts did not match the inclusion criteria, which resulted in identifying 68 and 76 full-text articles for fasting and starvation, respectively, to be included in the study, equaling 144 articles in total. Of these 144, an additional 57 full-text articles were excluded due to not being related to the objective, as described in the following PRISMA flow diagram (Figure 1). A total of 88 full-text studies remained for this review, with 37 addressing fasting and 50 addressing starvation. In addition, one article addressed both fasting and starvation (Figure 1).

Full-text articles were mapped to the metabolic impacts of each indicated endocrine hormone during fasting and starvation. For fasting, we mapped ten articles to cortisol, ten articles to glucagon, ten articles to insulin, and seven articles to thyroid hormones. For starvation, we mapped fifteen articles to cortisol, five articles to glucagon, eleven articles to insulin, and nineteen articles to thyroid hormones. One article, related to insulin, glucagon, and thyroid hormones, was mapped to both fasting and starvation. Table 2 summarizes the characteristics of the identified full-text scientific literature resources containing data that assisted us in meeting our objective and answering the study question. These characteristics were used during our search strategy as well. As shown in Table 2, the key findings from the reviewed articles indicate that while the serum levels of cortisol and glucagon are increased, the serum levels of insulin and T_3_ are decreased during both fasting and starvation. The different levels of these four hormones result in producing metabolic impacts to different extents, depending on the duration of food deprivation. More detailed data and results are presented in a supplementary table (Table S1) and in the results and discussions sections. The nature of the eligible studies was one book chapter, forty-two review studies, and forty-five clinical studies, which are indicated in Table S1 as well.

### 3.2. Release of Cortisol from Adrenal Glands during Fasting and Starvation

Cortisol is classified as a steroid hormone and is a major glucocorticoid hormone that is synthesized by the middle layer, the zona fasciculata, of the adrenal cortex. Cortisol plays an important role in the metabolism of macronutrients to support glucose homeostasis in response to increased energy demands [2]. Therefore, it is reasonable to explore its roles during fasting and starvation.

The circadian rhythm is a 24 h biological cycle that plays an important role on molecular, mental, and behavioral levels [43,108,109,110]. Cortisol is synthesized and released during each circadian rhythm cycle, which is based on the regulation of the HPA axis [111]. The HPA controls the level and magnitude of the corticotropin-releasing hormone (CRH), which is transported to the anterior pituitary via the hypothalamic–pituitary portal system to stimulate the release of adrenocorticotropic hormone (ACTH) [111]. The ACTH acts on the adrenal glands to synthesize and release cortisol into the bloodstream [111]. Of the cortisol released into circulation, about 80–90% are bound to transcortin (known also as corticosteroid-binding globulin (CBG), around 5–15% are bound to albumin, and around 5% are free cortisol (unbound). As a result, the CBG concentration plays an important role in regulating the accessibility of physiologically active cortisol [111].

There are a few stressful conditions in which the HPA axis is altered and cortisol release is enhanced [11]. These stressful conditions include low socioeconomic status, chronic work stress, anxiety, depression [112], and short-term [44] and long term [113] nutrient deprivation. Under healthy conditions and a non-fasting circadian rhythm, the release of cortisol follows the following pattern: a low level in the evening with an increasing level during the night followed by a peak in the early morning [114], in which cortisol reaches acrophase at 7:00–8:00 a.m. and declines until midnight [115,116]. It has been suggested that a nocturnal rise in HPA axis activity is a strategic and adaptive mechanism to prepare the human body for the upcoming daily requirement of energy demands [45,117,118].

The cortisol hormone is known to serve as a classic mediator of stress responses [11,12]. Factors such as fasting, stress, malnutrition, and anorexia increase cortisol levels [36,51,52,119,120]. In contrast to these findings, one study indicated that 48 h of starvation in 20 healthy women stressed steroid precursor production without leading to enhanced mineralocorticoid, glucocorticoid, or androgen production [50]. In a study on 208 healthy undergraduate students, their serum cortisol levels 1–3 h before a major examination indicated significantly high epinephrine, total cholesterol, high-density lipoprotein (HDL) cholesterol, low-density lipoprotein (LDL) cholesterol, and cortisol levels. The authors concluded that stress might have played an important role in the aforementioned cortisol release [121]. Studies on fasting have consistently indicated that cortisol’s circadian rhythm is disrupted during the month of Ramadan (i.e., levels are lower in the morning and higher in the evening compared to pre-Ramadan values) [122,123,124]. Yet, it has been reported that by one month after the end of Ramadan, cortisol levels had returned to normal pre-fasting ranges [125]. The authors concluded that the impact on circadian rhythm has to do with the stress of fasting and sleep deprivation on the body [125].

It has been questioned whether less severe caloric restriction (less than 800 kcal/day), for instance, during weight loss diets, also increases cortisol levels. To answer this question, a meta-analysis review study reported that starvation (2.5 days or longer) showed a very strong effect on the increase in serum cortisol, while less severe caloric restriction did not change the cortisol levels [11]. In another study, where 49 obese patients (body mass index (BMI) 32.2–67.1 kg/m^2^; 25 women and 24 men) underwent one-day fasting, it was indicated that the cortisol levels were increased [126]. While these results may be different in a daily fasting episode with no calorie intake for less than 24 h, they match the conclusion that no calorie intake, similar to starvation, increases cortisol levels [53,54]. There is an inverse relation between a low BMI, fat mass, and glucose and insulin concentrations and higher levels of cortisol, which is consistent with the finding that the release of cortisol is enhanced as an adaptation response to starvation threats [16]. It is possible that loss of the circadian rhythm causes hypercortisolism and chronic inflammation and increases the risk of chronic cardiometabolic disorders [123,124]. As a result, it has been suggested that there is a need to improve sleeping and feeding patterns during fasting in the month of Ramadan [123,124].

Boyar et al. measured the plasma levels of cortisol in 10 women who were experiencing AN [55]. Their clinical data indicated a normal pattern of the circadian cycle, but their 24 h mean plasma level was twice the upper limit of the normal level [55]. In addition, their data showed that the cortisol half-life was prolonged and the metabolic clearance rate was decreased [55]. The prolonged cortisol half-life parameter has been reported by other studies as well [56,127]. It is suggested that hypercortisolemia is an indicator of anorexia severity [128], and since cholesterol is used in the adrenal glands to produce cortisol, hypercholesterolemia (particularly LDL cholesterol) has been indicated in patients with AN [84]. It has been suggested that CBG levels were similar in the patients with anorexia and the controls [57], and the patients with AN experienced hypercortisolism [58,59]. It has been suggested that the high levels of cortisol are a result of a combination of a relative increase in cortisol secretion and a decrease in cortisol clearance [20]. It is well known that hypercortisolemia can have a severe effect on the body both physically and psychologically [105,129], which is associated with a series of clinical consequences such as low bone mineral density (BMD), depression and anxiety symptoms, and increased adrenal gland volumes [60,61,85,130,131]. While cortisol increases blood pressure and causes dyslipidemia [130], it also provides potent anti-inflammatory activities [132]. It is important to emphasize here that hypercortisolism may not show the same symptoms that Cushing’s syndrome presents since studies on patients with anorexia who experienced hypercortisolism, classic symptoms of Cushing’s syndrome have not been observed [100,133].

Cortisol inhibits the uptake of glucose from the circulation into skeletal muscle, which results in an increased blood glucose level, an effect caused by the decrease in the sensitivity of peripheral tissue to insulin [134]. In addition, cortisol stimulates the gluconeogenesis pathway [62]. Furthermore, cortisol increases the breakdown of skeletal muscle proteins (proteolysis), and released glucogenic and ketogenic amino acids produce glucose (gluconeogenesis) and ketone bodies (ketogenesis), respectively [2,3]. Due to the amino acid oxidation pathway, urea production (ureagenesis) is increased as well [3]. Furthermore, cortisol plays an important role in lipid metabolism by increasing the breakdown of triacylglycerols (lipolysis) into fatty acids and glycerol in adipocytes [2]. Produced fatty acids are used to provide ATP in order to support the energy expenditure of the gluconeogenesis pathway to synthesize glucose [3]. Similarly, released glycerol is utilized to serve as a gluconeogenic precursor [2].

Since glycogen is exhausted within the first 24 h of fasting, cortisol release can help the body have access to glucose beyond 24 h of starvation via gluconeogenic pathways [2,62]. It has been reported that there is a strong inverse relationship between adiposity in patients with AN and the serum concentration of alanine aminotransferase (ALT) [7,135]. ALT’s role in skeletal muscle is to remove the nitrogen group from the amino acid glutamate and add it to pyruvate to synthesize the glucogenic amino acid alanine. Alanine plays a central role in urea formation as it travels to the liver, where it donates its amino group to the hepatic α-ketoglutarate by the hepatic ALT to produce glutamate, which eventually enters the urea cycle to eliminate the toxic ammonium in the form of harmless urea [3]. The above inverse correlation between AN and ALT concentrations suggests an adaptive mechanism to assist the starved individuals in the prevention of the buildup of toxic ammonium, particularly during 2–3 days of starvation.

Because cortisol stimulates gluconeogenesis to maintain euglycemia during starvation, it serves as an endogenous antagonist of insulin [134], and it has been suggested that leptin may act directly on the adrenal gland by inhibiting cortisol secretion [49]. As reported previously, fasting results in reduced insulin secretion and increased glucagon and epinephrine release [3]. The release of the latter two hormones results in an increased level of blood glucose. This is a protective mechanism to maintain a normal blood glucose level by increasing hepatic glucose output during fasting. Therefore, individuals with severe AN may experience hypoinsulinemia [62]. While there are inconsistent reports about leptin levels during fasting [3], the levels of leptin are clearer during starvation. It has been reported that leptin changes in patients with AN partly affect neuroendocrine axes during starvation [136]. For instance, leptin inhibits the HPA axis both at the hypothalamic and possibly directly at the adrenal level, and a lower leptin level is associated with activation of the HPA axis in response to starvation [137]. In other words, leptin concentration is inversely related to ACTH and cortisol levels [138].

### 3.3. Release of Glucagon and Insulin from Pancreas during Fasting and Starvation

The pancreas has islets of Langerhans that produce a few short-peptide hormones with unique biochemical and/or physiologic actions. Glucagon and insulin are two pancreatic hormones with 29 and 51 amino acid residues, respectively. From the pancreas, both hormones travel to the liver via the portal vein [3]. It has been suggested that glucose, insulin, and glucagon are critical molecules that directly or indirectly influence the enzymes that regulate liver carbohydrate and fatty acid metabolism during the transition between the fed and the fasted state [70]. While glucagon’s release is stimulated by hypoglycemia, starvation, exercise, and protein-rich meals [64], insulin release is stimulated by a hyperglycemic condition [3]. The plasma level of glucagon is increased after 1 h of experiencing a hypoglycemic condition [13]. The concentration of insulin circulating in the blood is increased after a meal, reduced during fasting [2,3], and even further reduced during starvation [84,139]. It is suggested that beta cells adapt by adjusting the transcription of the insulin gene during fasting [73].

There are a few factors, such as circulating amino acids, fatty acids, and glucagon-like peptides, that regulate glucagon secretion [140]. One of the most potent mechanisms to stimulate glucagon secretion is experiencing low plasma glucose concentrations [65,66,67]. In order to support glucose homeostasis, glucagon inhibits glycogenesis and stimulates glycogenolysis and gluconeogenesis [71,72,141,142,143]. Similar to cortisol’s inhibitory effect on lipogenesis in adipocytes, glucagon inhibits lipogenesis as well by inactivating the first step in the fatty acid synthesis from carbohydrates [144]. In order to remedy the hypoglycemic episode, hepatic gluconeogenesis plays a critical role in the synthesis of glucose during a 36 to 48 h period of food deprivation [145]. It has been suggested that glucagon levels in patients with AN are higher compared to the control groups [81], and during fasting, glucagon promotes the transcription of the gluconeogenesis gene [68]. It has been shown that the glucose output via gluconeogenesis accounts for more than 90% of the total glucose production during a 40 h period of starvation in humans [146]. These results suggest that plasma glucagon levels are increased during starvation, particularly during 2–3 days of starvation.

Under fasting conditions, glucagon’s release is increased to activate AMP-dependent protein kinase A (PKA), which in turn triggers transcriptional activity, ultimately leading to an increased expression of gluconeogenic genes such as phosphoenolpyruvate carboxykinase (PEPCK) and glucose-6-phosphatase (G6Pase) to stimulate hepatic gluconeogenesis [46]. It is known that insulin plays an important role in the hormonal control of metabolic adaptation during fasting [90]. Contrary to the increased glucagon levels, insulin levels are lower during fasting [2,3] and in patients with AN [15,16,17,18,19,84,85,86,139], and insulin levels negatively correlate with cortisol levels [86]. It has been suggested that the synthesis of very low-density lipoprotein (VLDL), a triacylglycerol-rich particle, is increased in hepatocytes in response to reduced insulin action under fasting conditions [76]. It is suggested that Ramadan fasting results in a transient reduction in insulin sensitivity, which is compensated by an improved β-cell function with no significant change in glucose concentrations [37]. It has been reported that increased levels of glucagon and low levels of insulin augment lipolysis and ketogenesis [26]. As was previously reported [3], insulin stimulates protein synthetic machinery and inhibits proteolysis, resulting in a reduction in circulating amino acids. As one might expect, reduced insulin levels in patients with AN will result in an increased oxidation of glucose to prevent the storage of glucose [87]. In a study that included 92 patients with AN, serum amino acid levels indicated hyperaminoacidemia [63]. Indeed, it has been suggested that fasting does not decrease plasma amino acid levels, with the exception of the glucogenic amino acid alanine, which is used for gluconeogenesis [77]. In a study where eight healthy individuals underwent food deprivation, blood concentrations of branched chain amino acids (BCAAs, i.e., valine, isoleucine, and leucine) were high between 36 and 60 h of starvation, and glucagon levels were increased within the same timeframe [147]. While it is known that circulating amino acids play a central role during 2–3 days of starvation when the gluconeogenesis pathway is active [2,3], the relationship between BCAAs, glucagon, and fasting or starvation is not fully understood.

It has been reported that patients with AN experience significant changes in the pancreatic secretion of glucagon [82]. In a study, the plasma levels of glucagon and insulin were obtained from 26 patients suffering from AN [15]. The results indicated that while basal plasma glucagon levels were higher in patients with AN compared with the control group, the difference was not statistically significant [15]. Therefore, it has been suggested that, in chronic starvation caused by AN, insulin plays a major role while glucagon has minor importance [15]. In another study, it was suggested that during fasting, glucose levels and insulin concentrations were decreased and the levels of glucagon were increased, which in turn reduced hepatic glucokinase activity to maintain glucose homeostasis [69]. Contrary to the conclusion of many studies that glucagon concentrations are increased in patients with AN, there have been a few other studies that showed a lower glucagon concentration and an increased insulin sensitivity in patients with AN [92,93,148]. Regardless of the glucagon level variations in patients with AN, based on all reported data, it is safe to assume that glucose and insulin levels are reduced in AN patients.

Insulin plays an important role in adipose tissue metabolic pathways. Insulin enhances glucose uptake by adipose tissue and inhibits the activity of hormone-sensitive lipase, an intracellular enzyme that hydrolyzes triacylglycerol into glycerol and fatty acids [3]. A reduced ratio of insulin to glucagon during fasting and starvation enhances cyclic AMP levels in adipose tissue, thereby increasing the activity of the lipase enzyme [3].

### 3.4. Release of Thyroid Hormones from Thyroid Gland during Fasting and Starvation

Thyroid hormones, triiodothyronine (T_3_) and thyroxine (T_4_), are iodinated derivatives of the amino acid tyrosine and are synthesized and released from the thyroid gland [38]. Thyroid hormones’ receptors are found in almost all tissues and regulate a myriad of cellular responses [149]. Thyroid hormones’ production in the thyroid gland is regulated by the hypothalamic–pituitary–thyroid (HPT) axis, in which the hypothalamus secretes thyrotropin-releasing hormone (TRH) in response to a series of signals (cold, severe stress, etc.) [150]. TRH stimulates the anterior pituitary to secrete thyroid-stimulating hormone (TSH). TSH binds to its receptor, leading to thyroid hormone biosynthesis and the release of T_4_ and T_3_ from the thyroid follicle cells into the bloodstream. While more T_4_ than T_3_ is produced, T_4_ becomes converted to T_3_, mostly by peripheral deiodination. Consequently, T_4_ can be referred to as a prohormone that serves as a reservoir of T_3_ [151].

Approximately 80% of the thyroid hormones secreted by the thyroid gland are in the form of T_4_, and about 20% are in the form of T_3_ [94]. The most active thyroid hormone is T_3_ because it can bind to thyroid hormone receptors (THRs) to produce genomic effects [152]. Upon synthesis and release, T_3_ circulates in the blood and binds at the cellular level to THRs that act as transcription factors [153]. While it has not yet been fully understood, T_3_ produces nongenomic effects as well because, in a few signaling pathways, it does not modify gene transcription [151]. It is well known that T_3_ plays an important role in cell differentiation and organogenesis during fetal and childhood development, heart rate regulation, gastrointestinal (GI) motility, gluconeogenesis and glycogenolysis, glucose uptake, and maintaining thermogenic and metabolic homeostasis in adult individuals [153] (see Table 3 as well). It has been suggested that fasting [94] and starvation [17,22,23,24,25] reduce T_3_ levels in the periphery. It has also been reported that fasting downregulates the HPT axis, which represents an energy-saving mechanism during food deprivation [96]. During special conditions such as cold exposure, fasting, and/or infection, there is a need to change the thyroid status for adaptation [97,98]. Therefore, it is relevant to discuss the thyroid gland’s roles during fasting and starvation.

It has been reported that small changes may occur in TSH, free T_3_ (fT_3_), and free T_4_ (fT_4_) levels in healthy individuals during fasting [153]. For instance, when serum T_4_ levels increased, there was no significant change in the levels of T_3_ [154]. In a study on 58 healthy individuals aged 18–45 years who participated in a 24 h fasting period, both plasma fT_4_ concentrations and concentrations of a T_3_ metabolite, reverse T_3_ (rT_3_), increased on average by 8% and 16%, respectively [155]. In another study on 11 healthy men, a 56 h period of food deprivation resulted in a 40% increase in cortisol levels and a 50% suppression of mean TSH levels, with no clear link to whether the increased cortisol caused the reduction in the TSH levels [156]. It has been suggested that white adipose tissue (WAT) stores energy as triacylglycerols, and in obesity, it becomes dysfunctional and promotes a pro-inflammatory, hyperlipidemic, and insulin-resistant environment [157]. Comparatively, brown adipose tissue (BAT) specializes in heat production by utilizing fatty acid and glucose and in the expression of uncoupling protein 1 (UCP1) to dissipate energy as heat [158]. Both WAT and BAT are affected by T_3_. In BAT, it stimulates thermogenesis by increasing the UCP1 expression [158]. Comparatively, T_3_ promotes lipolysis in WAT by increasing the release of fatty acids [159] to meet the energy requirements of gluconeogenesis [3]. It is also known that thyroid hormones stimulate ATPases in skeletal muscle, which ultimately enhances energy expenditure [160]. Additionally, it is reported that, during fasting, fatty acids are partially oxidized by muscle and the liver to produce ketone bodies, which ultimately serve as fuel for the brain [75].

There is growing evidence that T_3_ levels are reduced in patients with AN [22,23,24,25,26,27,28,100,101,102,103]. While most of the AN studies were conducted in females, one study compared the impact of AN on both males and females and found that changes in the thyroid axis were the same in both genders [161]. In another study, the plasma levels of T_3_ indicated a small fluctuation within 24 h of fasting; however, they dropped sharply after 24 h of fasting and even more after 48 h of starvation [162]. While there is reduced peripheral deiodination of T_4_ to T_3_ [163], there have been suggestions that the conversion of T_4_ to rT_3_ is enhanced in fasting and starvation [20,24,26,38,155]. It has been suggested that the T_3_/T_4_ ratio is lower in AN compared to the control group [104], and T_3_ levels were more affected than T_4_ levels during starvation [101]. Reduced T_3_ hormone levels trigger symptoms associated with hypothyroidism, including bradycardia, hypothermia, hypotension, dry skin, and a slowed metabolic rate [164]. These symptoms usually resolve when patients with AN gain weight [164].

Decreased T_3_ levels have been observed during fasting [94,95], and it is suggested that, during the month of Ramadan, serum changes in thyroid hormones and TSH are minimal and do not affect the health of fasting individuals [165]. It has been reported that the normal sensitivity of peripheral tissues and TSH from pituitary thyrotrop in the anterior pituitary to the different circulating thyroid hormones is maintained in patients with AN, and reduced T_3_ is the only thyroid hormone that affects the metabolic state of AN patients [23]. However, it has been suggested that by refeeding with a mixed diet or predominantly carbohydrates, changes in serum T_3_ and rT_3_ caused by fasting are reversed [165].

There is a growing body of evidence that another endocrine hormone, leptin, which is primarily secreted by adipocytes [39,166], stimulates the TRH to enhance the production of thyroid hormones [167]. Patients with a leptin receptor mutation are hypothyroid with a delayed TSH response to TRH stimulation [99]. It has been suggested that leptin levels are linked to body mass, i.e., a lowered body fat mass leads to decreased serum leptin concentrations in AN [30,168], and decreased leptin levels result in reduced T_3_ levels during fasting and starvation [33,78,169]. It has been demonstrated that a 3-day starvation period results in an 80% reduction in leptin levels [29]. A reduction in leptin results in reduced TRH and TRH levels [167], possibly reducing T_3_ levels as well. While the exact mechanism remains unclear, the inhibitory impact on TSH was confirmed when an exogenous leptin dose normalized TSH levels in healthy lean men during a 3-day food deprivation period [29]. Additionally, there is no clear mechanism that explains how the reduced T_3_ results in a slow metabolic rate during starvation, as other metabolic factors, such as reduced lean body mass, a reduction in BAT activity, and reduced expression of UCP3, are involved [170]. Putting all these results together, one can predict that, during starvation, reduced leptin results in reduced T_3_, which in turn results in a reduced metabolic rate to preserve energy stores when there is food deprivation [171]. Obviously, a slowed metabolic rate benefits a starved individual only if other clinical complications of hypothyroidism do not occur. It is worth mentioning that the reduced T_3_ hormone levels in AN are suggested to be distinguished from secondary hypothyroidism [20].

### 3.5. Cortisol’s Roles during Time-Specific Food Deprivation

#### 3.5.1. Fasting: Food Deprivation within 24 h

As described earlier, both starvation and fasting affect and change the circadian rhythm of cortisol. It has been consistently shown that cortisol’s circadian rhythm is disrupted during fasting in the month of Ramadan, i.e., levels are lower in the morning and higher in the evening compared to the pre-Ramadan values [123,124]. However, it has also been reported that by one month after the end of Ramadan, cortisol levels had returned to their normal pre-fasting ranges [125]. Cortisol does not play a critical role in an acute hypoglycemic episode (because it does not produce an immediate response), and the stored glycogen in the liver and muscle will suffice to produce glucose during fasting [2,3,47]. An increase in cortisol levels inhibits the uptake of glucose from the circulation into peripheral tissues (skeletal muscle and adipose tissue), resulting in an increase in blood glucose level [2], an effect that is caused by the decrease in the sensitivity of peripheral tissue to insulin [135]. Since the insulin effect on peripheral tissues is compromised and the depletion of stored glycogen is underway, perhaps the most important role of cortisol during fasting is to promote glycogenolysis to maintain glucose homeostasis.

#### 3.5.2. Starvation: Food Deprivation beyond One Day

During day 2 of food deprivation, the stored glycogen is depleted [2,3,47]. Since the brain, erythrocytes, kidney medulla, lens and cornea, and testes are heavily dependent on having continuous access to glucose as an energy source [2], the high plasma level of cortisol plays an important adaptive role in promoting glucose homeostasis by providing sufficient glucose concentrations. Skeletal muscle is the primary reservoir for expendable proteins to not only release amino acids to feed protein synthetic machinery but also to support hepatic gluconeogenesis during starvation [172]. It has been reported that more than 80% of glucose comes from gluconeogenesis after 42 h of starvation [173], and fasting causes the activation of gluconeogenesis [47,48]. Cortisol and glucagon both promote gluconeogenesis to provide a minimum plasma level of glucose [83]. This adaptive process is carried out by breaking down expendable proteins in skeletal muscle to release glucogenic and ketogenic amino acids [3]. As a result, patients with AN may experience hyperaminoacidemia [63]. Glucogenic amino acids are used via gluconeogenesis to synthesize glucose [2,3]. Ketogenic amino acids are used to produce acetyl-CoA and ketone bodies in the liver that ultimately produce NADH to undergo oxidative phosphorylation in order to produce ATP for the brain [3]. Due to the oxidation of amino acids, one should expect a higher nitrogen excretion, which ultimately leads to a higher production of urea via the urea cycle in the liver [3]. It has been suggested that overnight fasting results in an increased activity of hormone-sensitive lipase in adipose tissue [174], which provides glycerol and free fatty acids to the circulation [79,175]. Hormone-sensitive lipase is highly expressed in adipose tissue and, to a lesser extent, in skeletal muscle [176]. It has been suggested that cortisol in physiological concentrations strongly stimulates lipolysis [177]. Since during food deprivation, insulin release is inhibited and cortisol release is stimulated, one might expect to see higher hormone-sensitive lipase activity during 2–3 days of starvation in order to support the energy expenditure of the gluconeogenesis pathway.

As mentioned earlier, ALT concentration is higher in patients with AN [178], and its role in skeletal muscle is to synthesize the glucogenic amino acid alanine, which ultimately will play a central role in ureagenesis [3], an adaptive mechanism to prevent the buildup of toxic ammonium, particularly during the early stage of starvation.

As the number of days of food deprivation increases, the physical and metabolic impacts of starvation become more visible. Skeletal muscle serves as a storage unit for expendable proteins that are utilized during starvation [3]. These non-essential proteins, however, will be depleted within a few days of starvation [3]. As a result, the transition from glucose metabolism to lipid metabolism is an important adaptive process to extend survival time [2]. The plasma level of free fatty acids is increased to twice the normal value in patients with AN [83]. In order to preserve and protect essential proteins from degradation and provide energy for vital functions, cortisol plays an important role in lipid metabolism. At this point, the brain gives up its addiction to glucose and adapts to use other molecules that ultimately can produce ATP molecules for the brain. It is believed that cortisol increases the breakdown of triacylglycerols (lipolysis) into fatty acids and glycerol [177], of which the latter molecule directly supports glucose output, whereas fatty acids’ oxidation serves as an alternative endogenous energy source [2].

Because cortisol stimulates gluconeogenesis to maintain euglycemia during starvation, it serves as an endogenous antagonist of leptin and insulin [62]. Therefore, individuals with severe AN experience hypoinsulinemia [85,179], and a low level of insulin augments lipolysis and ketogenesis [180]. In a large epidemiological study, the Epidemiology of Diabetes and Ramadan study, the authors demonstrated that daily fasting during the month of Ramadan increases the risk of severe hypoglycemia by 4.7-fold and 7.5-fold in patients with type 1 and type 2 diabetes mellitus, respectively, and even when they were treated with their antidiabetic agents [181], severe hypoglycemic episodes were reported [181,182]. However, other studies have shown that patients who used their antidiabetic agents did not experience hypoglycemic episodes during the month of Ramadan [183]. On the other hand, in a study conducted on women with both type 1 diabetes mellitus and AN, the mortality rate was 34.8%, i.e., at least a five-fold increase, compared with having diabetes type 1 or AN alone [184]. As a result, it is prudent to pay closer attention to diabetes complications whenever there is a warning sign of AN. It is worth mentioning that in a study conducted in Sweden by Ji et al. that focused on severe caloric restriction in individuals with AN, cortisol proved to be protective against the development of type 2 diabetes [185].

The above cortisol’s adaptive mechanisms will continue as long as the starved individual has fat mass to produce ketone bodies. It was shown that the expression of hypothalamic orexigenic neuropeptides was increased when mice received an infusion of ketone body (β-hydroxybutyrate) for 24 h, which resulted in an increased food intake [186]. This study was in mice and indicated the signaling role of the ketone body that can trigger hunger for exogenous macronutrients to supply the brain with glucose (as opposed to using the endogenous ketone body as an energy source) [186]. However, the ketone body’s orexigenic role in humans, particularly during fasting and starvation, remains unclear. It is important to emphasize here that pharmacologic treatment of AN to reduce cortisol levels is not recommended because it may result in further weight loss or promote adrenal insufficiency [187]. Table 2 summarizes the effects cortisol produces during fasting and starvation, and Table S1 provides more detailed information about cortisol’s specific effects.

### 3.6. Glucagon and Insulin’s Roles during Time-Specific Food Deprivation

#### 3.6.1. Fasting: Food Deprivation within 24 h

When blood glucose levels are low, glucagon is released to stimulate hunger [153,188]. On the other hand, insulin is released to suppress the feeling of hunger [188]. The role of insulin is to reduce excess glucose by promoting glycogen synthesis and lipogenesis in the liver [74] and to enhance the uptake of glucose by skeletal muscle and adipose tissue to synthesize glycogen and triacylglycerols, respectively [3].

In order to produce ATP molecules as an energy source and promote glucose homeostasis, one has to initiate the glycogenolysis pathway to release and supply glucose to the blood and tissues during fasting and starvation [2,75,134]. Both insulin and glucagon hormones play essential roles in glycogenolysis during the first day of food deprivation. Since insulin inhibits glycogenolysis in the liver and skeletal muscle [189], a reduced insulin level will support glucagon in stimulating glycogenolysis [2,3].

As was reported previously [3], plasma levels of glucagon and insulin are increased and reduced, respectively, during fasting. Glucagon inhibits glycogen synthase, the rate-limiting enzyme of glycogen metabolism, and activates glycogen phosphorylase *a* in the liver, triggering glycogenolysis [190]. As a result, glucose molecules are not stored or concentrated in the form of glycogen but are rather released from the tissues they originate from to supply energy. Because the liver plays a central role in maintaining a constant blood glucose level, glucose is used for hepatic glycolysis or transported to extrahepatic tissues by circulation [3].

Insulin levels begin to decrease during the early stages of food deprivation [3,19,35,38,50,73]. Since the body has enough glycogen during the first 24 h of food deprivation, perhaps the most profound impact of reduced insulin levels is to lower the cellular uptake of glucose by skeletal muscle and adipose tissue. It has been suggested that there are no correlations between a single day of fasting and the proteolysis of muscle proteins [191].

#### 3.6.2. Starvation: Food Deprivation beyond One Day

As was discussed earlier, between days 1 and 3 of starvation, expendable proteins in skeletal muscle become a major source of glucose. Reduced levels of insulin continue to favor the proteolysis of expendable proteins in skeletal muscle, producing glucogenic amino acids to support gluconeogenesis, a pathway that is also stimulated by glucagon [2,3]. It has been suggested that lipolysis, proteolysis, and protein oxidation increase significantly during a 60 h fasting period [80]. One can, however, predict that urea production will be enhanced here as well (similar to the effect of cortisol) as a result of amino acid oxidation that produces ammonia, which is further metabolized to urea through the urea cycle in the liver. In individuals who experience starvation, lower insulin sensitivity is associated with higher cholesterol levels [192]. Since glucagon and insulin counteract each other’s metabolic effects, it is suggested that this ratio determines their metabolic effects rather than their absolute plasma concentrations [193]. The reduced ratio of insulin to glucagon enhances PKA, which in turn activates adipose lipase by translocating hormone-sensitive lipase from the cytosol to lipid droplets to hydrolyze triacylglycerols into glycerol and fatty acids [3]. This enzymatic hydrolysis is imperative here for two reasons: (i) the gluconeogenesis pathway is expensive (requires four ATP molecules to produce one glucose molecule), and as a result, fatty acid β oxidation concurrently occurs to support the energy expenditure of gluconeogenesis; and (ii) glycerol release from triacylglycerols serves as a gluconeogenic substrate for the gluconeogenesis pathway [2].

During the second and third days of food deprivation, glucogenic substrates (glucogenic amino acids and any available glycerol from triacylglycerols) are utilized to provide intermediate substrates (α-ketoglutarate, succinyl-CoA, fumarate) for the citric acid cycle, with the goal to produce oxaloacetate, which ultimately, by the gluconeogenesis pathway, is converted into glucose [2,3]. The citric acid cycle becomes slower and slower as more and more oxaloacetate molecules are taken off the citric acid cycle to undergo the gluconeogenesis pathway [3]. This process saturates the entry of acetyl-CoA into the citric acid cycle, and the accumulated acetyl-CoA favors the synthesis of ketone bodies, which are mostly used by the brain as an alternative energy source [2,3]. Ketogenic amino acids are utilized to produce ketone bodies as well [3]. Putting all these metabolic outcomes together, one can predict that the fatty acid β oxidation, gluconeogenesis, and ketogenesis pathways are promoted during the early stages of starvation (two–three days) to promote metabolic homeostasis.

Similar to the effects of cortisol, when the number of days with food deprivation continues to extend, a reduced insulin/glucagon ratio promotes ketogenesis here as well. The plasma levels of glucagon and insulin were determined in a study on 26 patients who suffered from AN. The results indicated that while basal plasma glucagon levels were higher in patients with AN compared to the control group, the difference was not statistically significant [15]. On the other hand, the above study indicated that insulin levels were reduced. The authors suggested that in chronic starvation caused by AN, insulin has a major role, while glucagon’s role is minor [15]. The reduced ratio of insulin to glucagon continues to enhance the activity of hormone-sensitive lipase to hydrolyze triacylglycerols into glycerol and fatty acids [3]. This mechanism promotes fatty acid β oxidation in the hepatic mitochondria and generates acetyl-CoA [3]. As explained above, the excess of acetyl-CoA saturates the citric acid cycle, resulting in increased ketogenesis [2,3], which is utilized as an energy source for the brain. Table 2 summarizes the effects glucagon and insulin produce during fasting and starvation, and Table S1 provides more detailed information about the specific effects of these two hormones.

### 3.7. Thyroid Hormones’ Roles during Time-Specific Food Deprivation

#### 3.7.1. Fasting: Food Deprivation within 24 h

The most active thyroid hormone is T_3_, which is produced by the deiodination of T_4_ [152]. Basolo et al. demonstrated that after a 24 h fasting period in 58 euthyroid healthy individuals with normal glucose regulation, the plasma levels of both T_4_ and rT_3_ increased [155]. There are reports, however, that the changes in serum T_3_ and rT_3_ caused by fasting are reversed by refeeding with a mixed diet or predominantly carbohydrates in both fasting [165] and starvation conditions [194,195]. While during a one-day fasting period, refeeding takes place at the end of the daily fasting period, no refeeding takes place during starvation as the lack of access to food continues.

It is known that T_3_ stimulates hormone-sensitive lipase activity in adipose tissue and regulates triacylglycerol and cholesterol metabolism and lipoprotein homeostasis in the liver [114,153]. T_3_ has also been reported to increase hepatic glycogenolysis [153]. Since glycogenolysis is an important step during the first 24 h of food deprivation, one can expect to link reduced T_3_ levels to a reduced level of glycogenolysis.

#### 3.7.2. Starvation: Food Deprivation beyond One Day

Similar to the above fasting impacts, the plasma levels of T_3_ [22,23,24,25,26,27,28] and TSH concentrations decrease in patients with AN [26,106]. As mentioned earlier, the most important impact on starvation in this time frame is stimulation of fatty acid oxidation and gluconeogenesis. Both glucagon and cortisol stimulate gluconeogenesis from skeletal muscle’s expendable proteins and utilize fatty acid β oxidation to meet the energy expenditure of gluconeogenesis [2,3]. The release of leptin and T_3_’s stimulatory effect on thermogenesis in BAT and skeletal muscle by stimulating the expression of UCP1 and UCP3, respectively, is important to emphasize here as well. While lower insulin levels have not been linked to a lower fat or fat-free mass, leptin levels have been linked to fat mass [196]. In patients with AN and a reduced fat mass, the secretion of leptin was found to be reduced [197] and adiponectin levels were shown to be increased [88]. It has been demonstrated that patients with AN have significantly decreased plasma leptin and markedly increased plasma adiponectin levels [89], and the increased adiponectin levels may serve as a compensatory mechanism for insulin resistance in patients with AN [107]. It has been suggested that a 3-day food deprivation period significantly reduces leptin levels [29]. There is a growing body of evidence demonstrating decreased leptin levels result in reduced T_3_ levels during starvation [167] and fasting [99]. When levels of UCP1 and UCP3 are increased, a high amount of free fatty acids is oxidized to produce heat as opposed to producing ATP. Reduced levels of T_3_ and leptin during starvation reduce both UCP1 and UCP3 expressions [198]. Therefore, one might predict that lower T_3_ levels during starvation assist the body in preserving resources for cortisol and glucagon to produce ATP as opposed to producing heat by T_3_. One might, however, expect hypothermic episodes in the starved individuals during this time frame.

It has been reported that the level of T_3_ is reduced and the level of rT_3_ is increased during starvation in humans [28,38]. In addition, both DI activity and THR availability are reduced during starvation [199]. Perhaps most metabolic impacts from reduced T_3_ during starvation can be linked to the reduced activities that are mentioned in Table 3 [153]. Among those activities, reduced thermogenesis and lipolysis are effective in conserving fatty acid oxidation for ATP production and sparing any synthesized glucose for glucose-dependent tissues (brain, erythrocytes, testis, kidney medulla, lens and cornea, testes, skeletal muscles during exercise). Until recently, rT_3_ has been identified as an inactive metabolite of thyroid hormone metabolism [200]. There have been reports indicating that rT_3_ may produce unfavorable outcomes, including end-stage chronic kidney disease, acute myocardial infarction, hepatic diseases, and increased intensive care unit (ICU) mortality [201].

Since ketone bodies are an alternative energy source for the brain [2,3], the above hormonal release and adaptive mechanisms will continue during starvation as long as the starved individual has fats to produce ketone bodies [2,3]. Since the synthesis of glucose reduces by day 4 of starvation, the brain begins to use ketones from day 4 until all fatty acids have been oxidized [2]. Table 2 summarizes the effects thyroid hormones produce during fasting and starvation, and Table S1 provides more detailed information about their specific effects.

## 4. Discussions

### 4.1. Interpretation of Findings and Identified Areas for Future Research Studies

Fasting and starvation are two food deprivation conditions that affect the metabolism of endogenous macronutrients. While in some aspects they act similarly, in other facets they extend their impacts differently. The findings from the literature search indicated that endocrine hormones such as cortisol, glucagon, insulin, and T_3_ play key, yet different, roles when a human body encounters food deprivation. The question that guided the objective of this review study was the following: what are the adaptive stimulatory and inhibitory effects that cortisol, glucagon, insulin, and T_3_ hormones exert to support and/or promote metabolic homeostasis during fasting and starvation? In order to answer this question, we looked at the adaptive roles that the above hormones played in the metabolism of endogenous macronutrients.

Under healthy conditions and when there is no shortage of food, the circadian rhythms of cortisol, insulin, glucagon, and T_3_ are regulated to release these hormones at specific times within a 24 h cycle. For instance, the serum level of cortisol reaches its highest level by early morning (7 a.m.) to stimulate wakefulness and prepare the human body for the energy demands that humans encounter during the day [45,115,116,117,118]. Similarly, the serum level of insulin reaches its highest level at 5 PM to promote nutrient storage during the fed state and prepare the human body for subsequent energy production during sleep, which represents a fasting period [43].

It has been reported that during fasting and starvation, the circadian rhythm is shifted to the extent that the serum levels of cortisol are increased during the day and decreased during the night [122,123,124], which could be associated with a prolonged half-life and decreased metabolic clearance of cortisol [55]. To what extent a prolonged shift in cortisol’s circadian rhythm during food deprivation causes physiological consequences is unclear. What is clear is that the release of cortisol during food deprivation promotes metabolic homeostasis by reducing peripheral glucose uptake [2,134] and increasing glycogenolysis in both muscle and liver [2]. The impact of the high cortisol levels during starvation is even more profound when the glycogen is exhausted after 24 h of food deprivation. In other words, the increased cortisol levels stimulate proteolysis to produce glucogenic amino acids and promote gluconeogenesis to synthesize glucose [2] to support glucose homeostasis, at least during the early stages of starvation. It is possible that, as a result of amino acid oxidation during gluconeogenesis and an increased ALT concentration, ureagenesis increases during the early stages of starvation.

Under healthy conditions and when there is no food deprivation, the release of insulin and glucagon changes depending on a few conditions. For instance, glucagon release is stimulated by hypoglycemia, starvation, exercise, and protein-rich meals [64], and insulin release is stimulated by a hyperglycemic condition [3]. During fasting and starvation, when the blood glucose levels drop, both insulin and glucagon play critical roles in maintaining glucose homeostasis [2,3,134]. As indicated in Table 2, the reduced ratio of insulin to glucagon during fasting and starvation has multiple effects on the metabolism of macronutrients to promote glucose homeostasis. A few of these pancreatic effects were also seen when the cortisol levels were increased during food deprivation, i.e., a reduced insulin/glucagon ratio enhances glycogenolysis [2,3,134] and reduces the peripheral uptake of glucose [2,3] during fasting and enhances gluconeogenesis, proteolysis, lipolysis, ureagenesis, and ketogenesis during starvation (Table 2 and Table S1) [2,3,13,16,83,90]. In other words, increased cortisol levels and a reduced insulin/glucagon ratio produce similar metabolic outcomes. This raises the question of why the levels of three hormones, albeit from two different endocrine organs, are changed to produce similar metabolic outcomes during fasting and starvation. One answer is that cortisol’s synthesis and release do not occur fast enough to swiftly respond to a drop in blood glucose levels, and rather, its role is to reduce the insulin sensitivity of peripheral tissue to insulin [134], which will ultimately support glucose homeostasis. Another answer may be linked to cholesterol levels. Cholesterol synthesis is reduced during fasting but is increased during starvation [3,84]. During starvation, continuous lipolysis produces glycerol and fatty acid molecules. While glycerol, as a glucogenic molecule, supports gluconeogenesis during the first few days of starvation, the continuous fatty acid oxidation produces acetyl-CoA, which is used to support ketogenesis and cholesterol synthesis. Ketogenesis is a critical mechanism to supply ATP for the brain, and cholesterol is an essential molecule used in the biosynthesis of cortisol. Putting these biomolecular changes together, the increased cortisol levels seem to create an adaptation mechanism to assist individuals in surviving long-term food deprivation during starvation. It is, however, important to emphasize that hypercortisolemia can have a series of clinical consequences, such as low bone mineral density (BMD), depression and anxiety symptoms, and increased adrenal gland volumes [60,61,85,130,131]. Nevertheless, more studies are needed to understand the role of cholesterol during starvation in order to shed light on an adaptation mechanism that links cortisol, glucagon, insulin, and cholesterol with the metabolism of endogenous macronutrients.

As presented in Table 3, the most active thyroid hormone, T_3_, binds to its receptors to produce multiple cellular responses [153]. Similar to the HPA axis that regulates cortisol’s synthesis and release from adrenal glands, thyroid hormones’ production in the thyroid gland is regulated by the HPT axis [150]. It has been suggested that fasting and starvation reduce T_3_ levels in the periphery [2,28,94,155], which, for fasting, is a result of reduced TSH, deiodinase activity, or receptor availability [2,95,165].

One of the T_3_ roles during non-fasting conditions is to enhance glycogenolysis. As one might expect, reduced T_3_ during fasting may reduce glycogenolysis, which is in sharp contrast to what cortisol and glucagon hormones do during fasting. However, this mechanism represents an intriguing energy-saving mechanism during food deprivation by affecting thermogenesis. As mentioned earlier, T_3_ affects both WAT and BAT. In BAT, it stimulates thermogenesis by increasing UCP1 expression [158], and in WAT, it promotes lipolysis [159] to meet the energy requirement of gluconeogenesis [3]. The thermogenesis role of T_3_ may explain why glycogenolysis is prone to being reduced by T_3_ but is increased by cortisol and glucagon during fasting. In other words, a reduced level of T_3_ reduces glycogenolysis to avoid wasting energy as heat and instead leaves glycogen to undergo glycogenolysis by cortisol and glucagon during fasting, i.e., a classic crosstalk between these three hormones. Additionally, the fact that glycogenolysis is stimulated during the first day of food deprivation indicates that cortisol’s and glucagon’s stimulatory effects on glycogenolysis are higher than the inhibitory effect of T_3_ during food deprivation. This is an intriguing area that one needs to explore and study further in order to elucidate the impact of T_3_ on glycogenolysis during fasting.

It has been suggested that rT_3_ levels are increased when there is food deprivation [38,105,155], and rT_3_ may produce unfavorable clinical outcomes [201]. It is, however, unclear whether increased rT_3_ levels during fasting or starvation are linked to unfavorable clinical outcomes. This area warrants further studies to understand the role of rT3 during fasting and starvation. It is important to mention here that it is suggested that changes in thyroid hormone levels are a response to food deprivation and are not identified as secondary hypothyroidism [20]. Consequently, it may not be appropriate to treat AN patients with thyroid hormones.

It has been suggested that starvation results in a significant reduction in leptin levels, which results in reduced TSH and TRH [167,171], a pathway that was confirmed when the administration of an exogenous leptin normalized TSH levels during the food deprivation period [29,171]. These results indicate that suppressed TSH levels during food deprivation are mediated by leptin [171], and patients with AN show low levels of leptin, T_3_, and T_4_ [30]. The relationship between insulin and T_3_ is an intriguing observation as well. It is known that leptin and TSH have similar circadian rhythms (peak release around 1 a.m.) [43,171], and it is possible that both leptin and T_3_ affect thermogenesis through the same mechanism [167]. It has been reported that T_3_ increases β cell development to produce insulin and amylin hormones. These biochemical and molecular changes raise the critical question of whether reduced T_3_ levels during fasting and starvation play direct (or indirect) roles in reducing insulin levels. In other words, the fact that a reduced level of insulin is experienced during food deprivation is an outcome of a reduced T_3_ level, which in turn is a result of a reduced leptin level, with the ultimate goal of preserving energy stores when there is food deprivation. While it has been suggested that leptin does not affect the expression and/or activity of deiodinases (the enzymes that metabolize T_4_ and T_3_) or the levels of thyroxine-binding globulin (TBG, the protein that reduces free thyroid hormones) [29], there needs to be more research studies to illustrate a direct link between insulin, T_3_, and leptin.

### 4.2. Food Deprivation beyond 60 Days

An average body mass should provide fats for up to approximately two months to assist the starved individual in surviving the threat of starvation [202]. As a result, individuals who are trapped in a confined area with food deprivation will survive for up to 60 days, depending on access to water and their body mass. There have been many cases where individuals have survived food deprivation for a prolonged time frame. One clear example was when, in the summer of 2018, a 25-year-old assistant coach and 12 young soccer players, aged 11–16, were trapped in a cave in northern Thailand for nearly 18 days [203]. Upon rescue, none of the 13 individuals had any serious medical conditions. In other words, most metabolic complications and changes in neuroendocrine and neuropeptide levels normalize after recovery [204] and upon access to food and weight restoration. It is important, however, to clinically observe rescued individuals following the rescue process to ensure that their hormonal levels reverse to normal ranges after receiving adequate nutrients and care.

**Figure 2:**
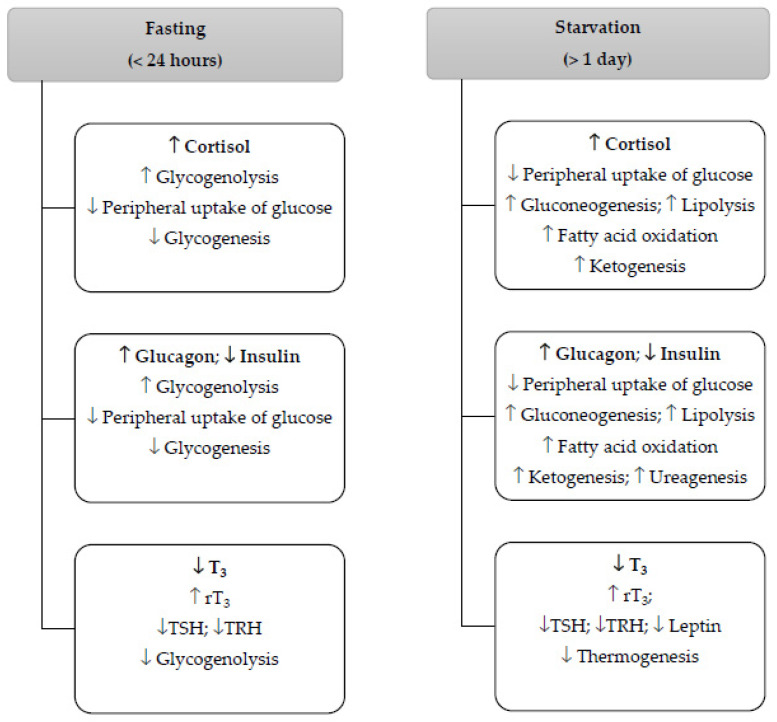
Effects of endocrine hormones on metabolism of endogenous macronutrients during fasting (<24 h) and starvation (>1 day). TRH: thyrotropin-releasing hormone; T_3_: triiodothyronine; rT_3_: reverse T_3_; TSH: thyroid-stimulating hormone; upward arrow (↑) indicates “increase” and downward arrow (↓) indicates “reduction” for a metabolic reaction during the indicated food deprivation. The data presented in this figure are extracted from eligible full-texts and also from other records through reference checking of the eligible full-texts.

Simply put, during days 2 and 3 of starvation, the proteolysis of expandable muscle proteins and gluconeogenesis pathways are critical to promoting glucose homeostasis, and by the fourth day of starvation, fatty acids are the major source of energy via the β oxidation pathway in the hepatic mitochondria [2]. The end result of this oxidative pathway is to produce many acetyl-CoA molecules [2,3,4]. The acetyl-CoA enters the citric acid cycle to interact with citrate and produce isocitrate, which, through a series of reactions, generates GTP, electron carrier NADH, and oxaloacetate [3,205]. The generation of too many acetyl-CoA molecules from the β oxidation of fatty acids saturates the citric acid cycle, resulting in the formation of ketone bodies (ketogenesis). As a result, in prolonged starvation (≥4 days) cases, ketone bodies [180,192,205], particularly acetoacetate and β-hydroxybutyrate, become a preferred and significant energy resource for the brain by producing NADH [2,3]. NADH is a valuable electron carrier molecule that, during oxidative phosphorylation, produces ATP molecules. This form of fatty acid oxidation and ketogenesis continues until the starved individual exhausts all of their fatty acids [2]. The fatty acid exhaustion, however, does not stop the body from producing the energy source molecule, ATP. At this late stage of starvation, which corresponds to approximately 2 months, the only source of ATP is proteins [2]. The degradation of these structural and functional proteins causes severe metabolic and enzymatic consequences, resulting in the loss of function of vital organs, leading to a fatal outcome. The brain constitutes only 2% of the total body mass but requires 20% of the body’s total energy expenditure at rest [2]. As a result, the brain continuously safeguards its own energy supply at the expense of all other organs, and thus has been referred to as “the selfish brain” [91,206]. However, from an evolutionary point of view, the brain serves the starved individual by extending survival time and maintaining its functional integrity, which assists in finding a way to survive. The adaptive stimulatory and inhibitory effects that cortisol, glucagon, insulin, and T_3_ hormones exert to promote metabolic homeostasis during fasting and starvation, respectively, are summarized in Figure 2.

### 4.3. Strengths and Limitations

This review study has several strengths. One overarching strength of this review study is that our literature search brings together findings that compare and contrast the metabolic impacts and outcomes during two different food deprivations, fasting and starvation. Another strength is that we have included a large number of studies and mapped the adaptive roles endocrine hormones play to assist humans in meeting daily energy demands during fasting and starvation. The review study has mapped the existing literature and identified a missing link between T_3_ and three other hormones, cortisol, glucagon, and insulin. Our findings depict relationships between different hormones and provide a rationale for the stimulatory and inhibitory mechanisms that endocrine hormones extend and exert to metabolize macronutrients during food deprivation. Lastly, our findings can serve as an educational resource to educate students from different health professions to maximize their understanding of the adaptive roles that endocrine hormones play during different forms of food deprivation.

This review study, however, has a few limitations. While the month of Ramadan represents a consistent fasting time to study (i.e., less than 24 h for healthy individuals), the studies were sparse, which resulted in reviewing eligible full texts, which used inconsistent fasting time frames in their studies. Additionally, the month of Ramadan is highly regarded and practiced around the world, particularly in the Middle East, and it is possible that there are studies in languages other than English that are missing from our study. We did not expect to find any starvation studies in humans (due to ethical reasons), and we used AN studies as the closest representation of starvation in humans. Similar to the above time frame inconsistency in fasting articles, AN studies also showed a variation regarding the time frame of starvation. Another limitation is that most patients with AN are adolescents or young women. Whether the metabolic outcomes would be different among men and women and whether a broader range of ages produces different metabolic outcomes remain to be seen in future research studies. Moreover, most of the included clinical studies were cross-sectional studies in which hormonal changes were measured at one point in time with limited follow-up procedures and impacts. Lastly, a short number of search terms and only one database (PubMed) were used, and we did not assess the risk of selection bias in our study, which may have led to a narrowed selection process of published data.

## 5. Conclusions

While two different food deprivations, fasting < 24 h and starvation > 1 day, produce a few similar metabolic outcomes, there are differences in how each of these two forms of food deprivation primes the human body to adapt and promote glucose and metabolic homeostasis. During both fasting and starvation, the serum levels of cortisol, glucagon, and rT_3_ are increased and the serum levels of insulin and T_3_ are reduced. The adaptive crosstalk between these endocrine hormones triggers metabolic homeostasis by inhibiting the peripheral uptake of glucose and glycogenesis and stimulating glycogenolysis, proteolysis, gluconeogenesis, lipolysis, fatty acid oxidation, ketogenesis, and ureagenesis pathways. Reduced T_3_ levels reduce glycogenolysis during fasting and decrease thermogenesis during starvation, which ultimately serves as an adaptive energy-saving mechanism during food deprivation. These inhibitory effects of T_3_ preserve macronutrient energy content, which, with support from the stimulatory effects of cortisol and glucagon on glycogenolysis, lipolysis, and ketogenesis, assist starved individuals in resisting the starvation threat and extending their survival time. Our findings indicate that the inhibitory roles of the T_3_ hormone during starvation play a major role in preserving energy by allowing cortisol and glucagon to utilize their metabolic effects to their full extent to metabolize endogenous macronutrients. Additionally, our findings depict a potential link between leptin and T_3_ in affecting the release of insulin during food deprivation. These findings indicate that endocrine hormones are subject to influencing each other at several metabolic pathways during fasting and starvation, and the integral and adaptive effects of endocrine hormones are an inherent survival mechanism during food deprivation, particularly during starvation.

## Figures and Tables

**Table 1:** Summary of physiological and biochemical similarities and differences between fasting and starvation.

	Fasting (Food Deprivation for <24 h)	Starvation (Food Deprivation for >1 Day)
Characteristics	Often follows a cyclic feeding/fasting pattern	Usually does not follow any pattern
	Temporary, usually short-term, partial or complete abstinence from food	Long-term or persistent food deprivation
	Exemplified by a religious commitment or a cultural belief; can be utilized for diagnostic and treatment purposes of various disease states	Exemplified by a hunger strike, drought, war, famine, natural disaster, and anorexia nervosa
Metabolic Effects on Humans [2,3,4,37,38,39]	Triggers a short-term metabolic adaptation	Challenges metabolic homeostasis
	Reduces peripheral glucose uptake/usage	Reduces peripheral glucose uptake/usage
	Triggers glycogenolysis	Triggers proteolysis and gluconeogenesis
	Spares essential proteins	Proteolysis of essential and expendable proteins
	Causes negligible ketogenesis	Triggers significant ketogenesis
	May provide health benefits	Leads to exhaustion of endogenous energy reserves

**Table 2:** A summary of identified scientific literature resources containing pertinent information on adaptive effects of endocrine hormones on human metabolism of endogenous macronutrients during fasting and starvation. More specific and detailed information is provided in a supplementary table (Table S1).

Endocrine Organ (Hormone)	Fasting or Starvation	Key Findings That Relate to the Study Question	Reference
Adrenal glands (cortisol)	Fasting	↑Cortisol	[29,32,43,44,45]
		↑Gluconeogenesis	[46]
		↓Peripheral tissue glucose uptake and utilization	[47]
		↑Glycogenolysis	[48]
		↓Leptin leading to ↑Cortisol	[49]
		*↓Cortisol*	[50]
	Starvation	↑Cortisol	[20,36,51,52,53,54,55,56,57,58,59,60,61]
		↑Gluconeogenesis	[62]
		↑Amino acids	[63] *
Pancreas (glucagon and insulin)	Fasting	↑Glucagon	[64,65,66,67,68,69]
		Fasting benefits	[37]
		↑Glycogenolysis	[70,71,72]
		↓Glycogenesis	[35]
		↓Insulin	[73,74]
		↑Glucagon ↓Insulin ↑Glycogenolysis ↑Lipolysis ↑Ketogenesis	[75]
		↑Gluconeogenesis ↑Amino acids oxidation ↑Ureagenesis	[76,77]
		↓Insulin ↑Cortisol ↓Leptin	[34,78]
		↑Lipolysis	[79]
		↑Proteolysis	[80]
	Starvation	↑Glucagon	[15,81,82]
		↑Lipolysis ↑Ketogenesis	[83]
		↓Insulin	[19,84,85,86,87,88,89]
		↑Fatty acid oxidation ↑Ketogenesis	[90]
		↑Lipolysis ↑Gluconeogenesis	[16]
		↑Competition for energy resources	[91]
		*↓Glucagon*	[92]
		*↑Insulin sensitivity*	[93]
	Fasting and Starvation	↑Glucagon ↓Insulin ↓T_3_	[38]
Thyroid gland (thyroid hormones)	Fasting	↓T_3_ ↓TSH ↓TRH	[94,95]
		↓TSH	[96]
		↓TRH	[97,98]
		Fasting’s benefits	[39]
		↓Leptin leading to ↓T_3_	[99]
	Starvation	↓T_3_	[17,22,23,24,25,27,28,100,101,102,103,104]
		↑rT_3_	[18,21,105]
		↓TSH	[33,106]
		↓TBG	[26]
		↓Leptin	[107]

* While no direct measurement of cortisol was undertaken, authors claim cortisol might support the metabolic basis for high plasma amino acids in patients with AN. Text in italics represents contradicting results. Upward arrow (↑) indicates “increase” and downward arrow (↓) indicates “reduction” in a hormone level or metabolic reaction during the indicated food deprivation. TRH: thyrotropin-releasing hormone; T_3_: triiodothyronine; rT_3_: reverse T_3_; TSH: thyroid-stimulating hormone; TBG: thyroxine-binding globulin.

**Table 3:** The effects of T_3_ hormone on different tissues and organs in healthy non-fasting/non-starving individuals.

Tissue/Organ	T_3_ Effect
GI	↑ gastric motility, which results in enhanced glucose absorption
Pancreas	↑ β cell development to produce insulin and amylin
Liver	↑ gluconeogenesis and glycogenolysis
WAT	↑ lipolysis by increasing the release of fatty acids to support gluconeogenesis.
BAT	↑ thermogenesis by increasing the UCP1 expression
Skeletal muscle	↑ glucose uptake; ↑ thermogenesis by increasing the UCP3 expression

Upward arrow (↑) indicates “increase”; BAT: brown adipose tissue; GI: gastrointestinal; WAT: white adipose tissue; UCP: uncoupling protein.

## Data Availability

Data are contained within the article.

## Supplementary Materials

The following supporting information can be downloaded at: https://www.mdpi.com/article/10.3390/metabo14060336/s1, Table S1: Identified scientific literature resources containing pertinent information on adaptive effects of endocrine hormones on human metabolism of endogenous macronutrients during fasting and starvation

## References

[1] Guyenet S.J., Schwartz M.W. (2012). Clinical review: Regulation of food intake, energy balance, and body fat mass: Implications for the pathogenesis and treatment of obesity. J. Clin. Endocrinol. Metab..

[2] McGrath K.H., Haller W., Bines J.E. (2023). Starvation and Fasting: Biochemical Aspects. Encyclopedia of Human Nutrition.

[3] Karimi R., Cleven A., Elbarbry F., Hoang H. (2021). The Impact of Fasting on Major Metabolic Pathways of Macronutrients and Pharmacokinetics Steps of Drugs. Eur. J. Drug Metab. Pharmacokinet..

[4] Anton S.D., Moehl K., Donahoo W.T., Marosi K., Lee S.A., Mainous A.G., Leeuwenburgh C., Mattson M.P. (2018). Flipping the Metabolic Switch: Understanding and Applying the Health Benefits of Fasting. Obesity.

[5] Neale J., Hudson L.D. (2020). Anorexia nervosa in adolescents. Br. J. Hosp. Med..

[6] (2013). American Psychiatric Association Diagnostic and Statistical Manual of Mental Disorders.

[7] Miller K.K., Grinspoon S.K., Ciampa J., Hier J., Herzog D., Klibanski A. (2005). Medical findings in outpatients with anorexia nervosa. Arch. Intern. Med..

[8] Steinhausen H.C. (2002). The outcome of anorexia nervosa in the 20th century. Am. J. Psychiatry.

[9] Lucas A.R., Beard C.M., O’Fallon W.M., Kurland L.T. (1991). 50-year trends in the incidence of anorexia nervosa in Rochester, Minn.: A population-based study. Am. J. Psychiatry.

[10] Sheldon J.H. (1937). Anorexia nervosa with special reference to the physical constitution. Lancet.

[11] Nakamura Y., Walker B.R., Ikuta T. (2016). Systematic review and meta-analysis reveals acutely elevated plasma cortisol following fasting but not less severe calorie restriction. Stress.

[12] Cay M., Ucar C., Senol D., Cevirgen F., Ozbag D., Altay Z., Yildiz S. (2018). Effect of increase in cortisol level due to stress in healthy young individuals on dynamic and static balance scores. North Clin. Istanb..

[13] Rix I., Nexøe-Larsen C., Bergmann N.C., Lund A., Knop F.K., Feingold K.R., Anawalt B., Blackman M.R., Boyce A., Chrousos G., Corpas E., de Herder W.W., Dhatariya K., Dungan K., Hofland J. (2000). Glucagon Physiology. Endotext.

[14] Besse-Patin A., Jeromson S., Levesque-Damphousse P., Secco B., Laplante M., Estall J.L. (2019). PGC1A regulates the IRS1:IRS2 ratio during fasting to influence hepatic metabolism downstream of insulin. Proc. Natl. Acad. Sci. USA.

[15] Blickle J.F., Reville P., Stephan F., Meyer P., Demangeat C., Sapin R. (1984). The role of insulin, glucagon and growth hormone in the regulation of plasma glucose and free fatty acid levels in anorexia nervosa. Horm. Metab. Res..

[16] Misra M., Klibanski A. (2014). Endocrine consequences of anorexia nervosa. Lancet Diabetes Endocrinol..

[17] Schreiber W., Schweiger U., Werner D., Brunner G., Tuschl R.J., Laessle R.G., Krieg J.C., Fichter M.M., Pirke K.M. (1991). Circadian pattern of large neutral amino acids, glucose, insulin, and food intake in anorexia nervosa and bulimia nervosa. Metabolism.

[18] de Rosa G., Corsello S.M., de Rosa E., Della Casa S., Ruffilli M.P., Grasso P., Pasargiklian E. (1983). Endocrine study of anorexia nervosa. Exp. Clin. Endocrinol..

[19] Fonseca V., Ball S., Marks V., Havard C.W. (1991). Hypoglycaemia associated with anorexia nervosa. Postgrad. Med. J..

[20] Douyon L., Schteingart D.E. (2002). Effect of obesity and starvation on thyroid hormone, growth hormone, and cortisol secretion. Endocrinol. Metab. Clin. N. Am..

[21] Curran-Celentano J., Erdman J.W., Nelson R.A., Grater S.J. (1985). Alterations in vitamin A and thyroid hormone status in anorexia nervosa and associated disorders. Am. J. Clin. Nutr..

[22] Komaki G., Tamai H., Mukuta T., Kobayashi N., Mori K., Nakagawa T., Kumagai L.F. (1992). Alterations in endothelium-associated proteins and serum thyroid hormone concentrations in anorexia nervosa. Br. J. Nutr..

[23] Bannai C., Kuzuya N., Koide Y., Fujita T., Itakura M., Kawai K., Yamashita K. (1988). Assessment of the relationship between serum thyroid hormone levels and peripheral metabolism in patients with anorexia nervosa. Endocrinol. JPN.

[24] Leslie R.D., Isaacs A.J., Gomez J., Raggatt P.R., Bayliss R. (1978). Hypothalamo-pituitary-thyroid function in anorexia nervosa: Influence of weight gain. Br. Med. J..

[25] Moore R., Mills I.H. (1979). Serum T3 and T4 levels in patients with anorexia nervosa showing transient hyperthyroidism during weight gain. Clin. Endocrinol..

[26] Tamai H., Mori K., Matsubayashi S., Kiyohara K., Nakagawa T., Okimura M.C., Walter R.M., Kumagai L.F., Nagataki S. (1986). Hypothalamic-pituitary-thyroidal dysfunctions in anorexia nervosa. Psychother. Psychosom..

[27] Moshang T., Parks J.S., Baker L., Vaidya V., Utiger R.D., Bongiovanni A.M., Snyder P.J. (1975). Low serum triiodothyronine in patients with anorexia nervosa. J. Clin. Endocrinol. Metab..

[28] de Rosa G., Della Casa S., Corsello S.M., Ruffilli M.P., de Rosa E., Pasargiklian E. (1983). Thyroid function in altered nutritional state. Exp. Clin. Endocrinol..

[29] Chan J.L., Heist K., DePaoli A.M., Veldhuis J.D., Mantzoros C.S. (2003). The role of falling leptin levels in the neuroendocrine and metabolic adaptation to short-term starvation in healthy men. J. Clin. Investig..

[30] Blüher S., Mantzoros C.S. (2004). The Role of Leptin in Regulating Neuroendocrine Function in Humans. J. Nutr..

[31] Kuo T., Harris C.A., Wang J.C. (2013). Metabolic functions of glucocorticoid receptor in skeletal muscle. Mol. Cell. Endocrinol..

[32] Koutkia P., Canavan B., Johnson M.L., DePaoli A., Grinspoon S. (2003). Characterization of leptin pulse dynamics and relationship to fat mass, growth hormone, cortisol, and insulin. Am. J. Physiol. Endocrinol. Metab..

[33] Reinehr T., Isa A., de Sousa G., Dieffenbach R., Andler W. (2008). Thyroid hormones and their relation to weight status. Horm. Res..

[34] Popovic V., Duntas L.H. (2005). Leptin TRH and ghrelin: Influence on energy homeostasis at rest and during exercise. Horm. Metab. Res..

[35] Klover P.J., Mooney R.A. (2004). Hepatocytes: Critical for glucose homeostasis. Int. J. Biochem. Cell Biol..

[36] Amorim T., Khiyami A., Latif T., Fazeli P.K. (2023). Neuroendocrine adaptations to starvation. Psychoneuroendocrinology.

[37] Vardarli M.C., Hammes H.P., Vardarli İ. (2014). Possible metabolic impact of Ramadan fasting in healthy men. Turk. J. Med. Sci..

[38] Martinez B., Ortiz R.M. (2017). Thyroid Hormone Regulation and Insulin Resistance: Insights From Animals Naturally Adapted to Fasting. Physiology.

[39] Das E., Moon J.H., Lee J.H., Thakkar N., Pausova Z., Sung H.K. (2018). Adipose Tissue and Modulation of Hypertension. Curr. Hypertens. Rep..

[40] Peters M.D., Godfrey C.M., Khalil H., McInerney P., Parker D., Soares C.B. (2015). Guidance for conducting systematic scoping reviews. Int. J. Evid. Based Healthc..

[41] Aromataris E., Riitano D. (2014). Constructing a search strategy and searching for evidence. Am. J. Nurs..

[42] Page M.J., McKenzie J.E., Bossuyt P.M., Boutron I., Hoffmann T.C., Mulrow C.D., Shamseer L., Tetzlaff J.M., Akl E.A., Brennan S.E. (2021). The PRISMA 2020 Statement: An Updated Guideline for Reporting Systematic Reviews. Syst. Rev..

[43] Gamble K.L., Berry R., Frank S.J., Young M.E. (2014). Circadian clock control of endocrine factors. Nat. Rev. Endocrinol..

[44] Bergendahl M., Iranmanesh A., Pastor C., Evans W.S., Veldhuis J.D. (2000). Homeostatic joint amplification of pulsatile and 24-hour rhythmic cortisol secretion by fasting stress in midluteal phase women: Concurrent disruption of cortisol-growth hormone, cortisol-luteinizing hormone, and cortisol-leptin synchrony. J. Clin. Endocrinol. Metab..

[45] Yu H., Xia F., Lam K.S., Wang Y., Bao Y., Zhang J., Gu Y., Zhou P., Lu J., Jia W. (2011). Circadian rhythm of circulating fibroblast growth factor 21 is related to diurnal changes in fatty acids in humans. Clin. Chem..

[46] Oh K.J., Han H.S., Kim M.J., Koo S.H. (2013). CREB and FoxO1: Two transcription factors for the regulation of hepatic gluconeogenesis. BMB Rep..

[47] Dimitriadis G.D., Maratou E., Kountouri A., Board M., Lambadiari V. (2021). Regulation of Postabsorptive and Postprandial Glucose Metabolism by Insulin-Dependent and Insulin-Independent Mechanisms: An Integrative Approach. Nutrients.

[48] Oh K.J., Han H.S., Kim M.J., Koo S.H. (2013). Transcriptional regulators of hepatic gluconeogenesis. Arch. Pharm. Res..

[49] Casanueva F.F., Dieguez C. (1999). Neuroendocrine regulation and actions of leptin. Front. Neuroendocrinol..

[50] Magyar B.P., Santi M., Sommer G., Nuoffer J.M., Leichtle A., Grössl M., Fluck C.E. (2022). Short-Term Fasting Attenuates Overall Steroid Hormone Biosynthesis in Healthy Young Women. J. Endocr. Soc..

[51] Paszynska E., Dmitrzak-Weglarz M., Tyszkiewicz-Nwafor M., Slopien A. (2016). Salivary alpha-amylase, secretory IgA and free cortisol as neurobiological components of the stress response in the acute phase of anorexia nervosa. World J. Biol. Psychiatry..

[52] Herpertz S., Albers N., Wagner R., Pelz B., Köpp W., Mann K., Blum W., Senf W., Hebebrand J. (2000). Longitudinal changes of circadian leptin, insulin and cortisol plasma levels and their correlation during refeeding in patients with anorexia nervosa. Eur. J. Endocrinol..

[53] Wassif W.S., Ross A.R. (2013). Steroid metabolism and excretion in anorexia nervosa. Vitam. Horm..

[54] Thavaraputta S., Ungprasert P., Witchel S.F., Fazeli P.K. (2023). Anorexia nervosa and adrenal hormones: A systematic review and meta-analysis. Eur. J. Endocrinol..

[55] Boyar R.M., Hellman L.D., Roffwarg H., Katz J., Zumoff B., O’Connor J., Bradlow H.L., Fukushima D.K. (1977). Cortisol secretion and metabolism in anorexia nervosa. N. Engl. J. Med..

[56] Takahara J., Hosogi H., Yunoki S., Hashimoto K., Uneki T. (1976). Hypothalamic pituitary adrenal function in patients with anorexia nervosa. Endocrinol. Jpn..

[57] Gwirtsman H.E., Kaye W.H., George D.T., Jimerson D.C., Ebert M.H., Gold P.W. (1989). Central and peripheral ACTH and cortisol levels in anorexia nervosa and bulimia. Arch. Gen. Psychiatry.

[58] Casper R.C., Chattertonm R.T., Davis J.M. (1979). Alterations in serum cortisol and its binding characteristics in anorexia nervosa. J. Clin. Endocrinol. Metab..

[59] Elegido A., Gheorghe A., Sepúlveda A.R., Andrés P., Díaz-Prieto L.E., Graell M., Marcos A., Nova E. (2019). Adipokines, cortisol and cytokine alterations in recent onset anorexia nervosa. A case-control study. Endocrinol. Diabetes Nutr..

[60] Gold P.W., Gwirtsman H., Avgerinos P.C., Nieman L.K., Gallucci W.T., Kaye W., Jimerson D., Ebert M., Rittmaster R., Loriaux D.L. (1986). Abnormal hypothalamic-pituitary-adrenal function in anorexia nervosa. Pathophysiologic mechanisms in underweight and weight-corrected patients. N. Engl. J. Med..

[61] Salisbury J.J., Mitchell J.E. (1991). Bone mineral density and anorexia nervosa in women. Am. J. Psychiatry.

[62] Misra M., Klibanski A. (2010). Neuroendocrine consequences of anorexia nervosa in adolescents. Endocr. Dev..

[63] Moyano D., Vilaseca M.A., Artuch R., Lambruschini N. (1998). Plasma amino acids in anorexia nervosa. Eur. J. Clin. Nutr..

[64] Goldstein I., Hager G.L. (2018). The Three Ds of Transcription Activation by Glucagon: Direct, Delayed, and Dynamic. Endocrinology.

[65] Andersen D.B., Holst J.J. (2022). Peptides in the regulation of glucagon secretion. Peptides.

[66] Saltiel A.R. (2016). Insulin Signaling in the Control of Glucose and Lipid Homeostasis. Handb. Exp. Pharmacol..

[67] Nakata M., Yada T. (2007). PACAP in the glucose and energy homeostasis: Physiological role and therapeutic potential. Curr. Pharm. Des..

[68] Zhang L., Yao W., Xia J., Wang T., Huang F. (2019). Glucagon-Induced Acetylation of Energy-Sensing Factors in Control of Hepatic Metabolism. Int. J. Mol. Sci..

[69] Massa M.L., Gagliardino J.J., Francini F. (2011). Liver glucokinase: An overview on the regulatory mechanisms of its activity. IUBMB Life.

[70] van den Berghe G. (1991). The role of the liver in metabolic homeostasis: Implications for inborn errors of metabolism. J. Inherit. Metab. Dis..

[71] Taborsky G.J. (2010). The physiology of glucagon. J. Diabetes Sci. Technol..

[72] Sharabi K., Tavares C.D.J., Puigserver P. (2019). Regulation of Hepatic Metabolism, Recent Advances, and Future Perspectives. Curr. Diab Rep..

[73] Docherty K., Clark A.R. (1994). Nutrient regulation of insulin gene expression. FASEB J..

[74] Habegger K.M. (2022). Cross Talk Between Insulin and Glucagon Receptor Signaling in the Hepatocyte. Diabetes.

[75] Ahima R.S. (2006). Adipose tissue as an endocrine organ. Obesity.

[76] Kamagate A., Dong H.H. (2008). FoxO1 integrates insulin signaling to VLDL production. Cell Cycle.

[77] Bröer S., Bröer A. (2017). Amino acid homeostasis and signaling in mammalian cells and organisms. Biochem. J..

[78] Ahima R.S., Qi Y., Singhal N.S. (2006). Adipokines that link obesity and diabetes to the hypothalamus. Prog. Brain Res..

[79] Daval M., Foufelle F., Ferré P. (2006). Functions of AMP-activated protein kinase in adipose tissue. J. Physiol..

[80] Carlson M.G., Snead W.L., Campbell P.J. (1994). Fuel and energy metabolism in fasting humans. Am. J. Clin. Nutr..

[81] Heruc G.A., Little T.J., Kohn M.R., Madden S., Clarke S.D., Horowitz M., Feinle-Bisset C. (2018). Effects of starvation and short-term refeeding on gastric emptying and postprandial blood glucose regulation in adolescent girls with anorexia nervosa. Am. J. Physiol. Endocrinol. Metab..

[82] Kumai M., Tamai H., Fujii S., Nakagawa T., Aoki T.T. (1988). Glucagon secretion in anorexia nervosa. Am. J. Clin. Nutr..

[83] Casper R.C. (1996). Carbohydrate metabolism and its regulatory hormones in anorexia nervosa. Psychiatry Res..

[84] Weinbrenner T., Züger M., Jacoby G.E., Herpertz S., Liedtke R., Sudhopm T., Gouni-Berthold I., Axelson M., Berthold H.K. (2004). Lipoprotein metabolism in patients with anorexia nervosa: A case-control study investigating the mechanisms leading to hypercholesterolaemia. Br. J. Nutr..

[85] Misra M., Miller K.K., Almazan C., Ramaswamy K., Lapcharoensap W., Worley M., Neubauer G., Herzog D.B., Klibanski A. (2004). Alterations in cortisol secretory dynamics in adolescent girls with anorexia nervosa and effects on bone metabolism. J. Clin. Endocrinol. Metab..

[86] Mocanu V., Vergely N., Voitellier P., Rachidi-Kousa A., Estour B. (2003). Correlations between carbohydrate metabolism and corticotrop axis parameters in anorexia nervosa. Pathophysiology.

[87] Franssila-Kallunki A., Rissanen A., Ekstrand A., Eriksson J., Saloranta C., Widén E., Schalin-Jäntti C., Groop L. (1991). Fuel metabolism in anorexia nervosa and simple obesity. Metabolism.

[88] Tural U., Iosifescu D.V. (2022). Adiponectin in anorexia nervosa and its modifiers: A meta-regression study. Int. J. Eat. Disord..

[89] Dostálová I., Smitka K., Papežová H., Kvasnicková H., Nedvídková J. (2007). Increased insulin sensitivity in patients with anorexia nervosa: The role of adipocytokines. Physiol. Res..

[90] Ho K.Y., Veldhuis J.D., Johnson M.L., Furlanetto R., Evans W.S., Alberti K.G., Thorner M.O. (1988). Fasting enhances growth hormone secretion and amplifies the complex rhythms of growth hormone secretion in man. J. Clin. Investig..

[91] Fehm H.L., Kern W., Peters A. (2006). The selfish brain: Competition for energy resources. Prog. Brain Res..

[92] Alderdice J.T., Dinsmore W.W., Buchanan K.D., Adams C. (1985). Gastrointestinal hormones in anorexia nervosa. J. Psychiatr. Res..

[93] Ilyas A., Hübel C., Stahl D., Stadler M., Ismail K., Breen G., Treasure J., Kan C. (2019). The metabolic underpinning of eating disorders: A systematic review and meta-analysis of insulin sensitivity. Mol. Cell. Endocrinol..

[94] van der Spek A.H., Fliers E., Boelen A. (2017). The classic pathways of thyroid hormone metabolism. Mol. Cell. Endocrinol..

[95] Iwen K.A., Oelkrug R., Brabant G. (2018). Effects of thyroid hormones on thermogenesis and energy partitioning. J. Mol. Endocrinol..

[96] Boelen A., Wiersinga W.M., Fliers E. (2008). Fasting-induced changes in the hypothalamus-pituitary-thyroid axis. Thyroid.

[97] Lechan R.M., Fekete C. (2006). The TRH neuron: A hypothalamic integrator of energy metabolism. Prog. Brain Res..

[98] Fekete C., Lechan R.M. (2007). Negative feedback regulation of hypophysiotropic thyrotropin-releasing hormone (TRH) synthesizing neurons: Role of neuronal afferents and type 2 deiodinase. Front. Neuroendocrinol..

[99] Janeckova R. (2001). The role of leptin in human physiology and pathophysiology. Physiol. Res..

[100] Støving R.K., Hangaard J., Hansen-Nord M., Hagen C. (1999). A review of endocrine changes in anorexia nervosa. J. Psychiatr. Res..

[101] Croxson M.S., Ibbertson H.K. (1977). Low serum triiodothyronine (T3) and hypothyroidism in anorexia nervosa. J. Clin. Endocrinol. Metab..

[102] Capo-chichi C.D., Guéant J.L., Lefebvre E., Bennani N., Lorentz E., Vidailhet C., Vidailhet M. (1999). Riboflavin and riboflavin-derived cofactors in adolescent girls with anorexia nervosa. Am. J. Clin. Nutr..

[103] Onur S., Haas V., Bosy-Westphal A., Hauer M., Paul T., Nutzinger D., Klein H., Müller M.J. (2005). L-tri-iodothyronine is a major determinant of resting energy expenditure in underweight patients with anorexia nervosa and during weight gain. Eur. J. Endocrinol..

[104] Miyai K., Yamamoto T., Azukizawa M., Ishibashi K., Kumahara Y. (1975). Serum thyroid hormones and thyrotropin in anorexia nervosa. J. Clin. Endocrinol. Metab..

[105] Schorr M., Miller K.K. (2017). The endocrine manifestations of anorexia nervosa: Mechanisms and management. Nat. Rev. Endocrinol..

[106] Kiyohara K., Tamai H., Takaichi Y., Nakagawa T., Kumagai L.F. (1989). Decreased thyroidal triiodothyronine secretion in patients with anorexia nervosa: Influence of weight recovery. Am. J. Clin. Nutr..

[107] Pannacciulli N., Vettor R., Milan G., Granzotto M., Catucci A., Federspil G., De Giacomo P., Giorgino R., De Pergola G. (2003). Anorexia nervosa is characterized by increased adiponectin plasma levels and reduced nonoxidative glucose metabolism. J. Clin. Endocrinol. Metab..

[108] Manoogian E.N.C., Panda S. (2017). Circadian rhythms, time-restricted feeding, and healthy aging. Ageing Res. Rev..

[109] Loudon A.S.I. (2012). Circadian biology: A 2.5 billion year old clock. Curr. Biol..

[110] Edgar R.S., Green E.W., Zhao Y., Van Ooijen G., Olmedo M., Qin X., Xu Y., Pan M., Valekunja U.K., Feeney K.A. (2012). Peroxiredoxins are conserved markers of circadian rhythms. Nature.

[111] Ramamoorthy S., Cidlowski J.A. (2016). Corticosteroids: Mechanisms of Action in Health and Disease. Rheum. Dis. Clin. N. Am..

[112] Kann P.H., Münzel M., Hadji P., Daniel H., Flache S., Nyarango P., Wilhelm A. (2015). Alterations of cortisol homeostasis may link changes of the sociocultural environment to an increased diabetes and metabolic risk in developing countries: A prospective diagnostic study performed in cooperation with the Ovahimba people of the Kunene region/northwestern Namibia. J. Clin. Endocrinol. Metab..

[113] Pasiakos S.M., Caruso C.M., Kellogg M.D., Kramer F.M., Lieberman H.R. (2011). Appetite and Endocrine Regulators of Energy Balance After 2 Days of Energy Restriction: Insulin, Leptin, Ghrelin, and DHEA-S. Obesity.

[114] Molina P. (2023). Endocrine Physiology.

[115] Dickmeis T. (2009). Glucocorticoids and the circadian clock. J. Endocrinol..

[116] Morris C.J., Aeschbach D., Scheer F.A. (2012). Circadian system, sleep and endocrinology. Mol. Cell. Endocrinol..

[117] Born J., Hansen K., Marshall L., Mo¨lle M., Fehm H.L. (1999). Timing the end of nocturnal sleep. Nature.

[118] Benedict C., Kern W., Schmid S.M., Schultes B., Born J., Hallschmid M. (2009). Early morning rise in hypothalamic-pituitary-adrenal activity: A role for maintaining the brain’s energy balance. Psychoneuroendocrinology.

[119] Beer S.F., Bircham P.M.M., Bloom S.R., Clark P.M., Hales C.N., Hughes C.M., Jones C.T., Marsh D.R., Raggatt P.R., Findlay A.L.R. (1989). The effect of a 72-h fast on plasma levels of pituitary, adrenal, thyroid, pancreatic and gastrointestinal hormones in healthy men and women. J. Endocrinol..

[120] Vance M.L., Thorner M.O. (1989). Fasting alters pulsatile and rhythmic cortisol release in normal man. J. Clin. Endocrinol. Metab..

[121] Maduka I.C., Neboh E.E., Ufelle S.A. (2015). The relationship between serum cortisol, adrenaline, blood glucose and lipid profile of undergraduate students under examination stress. Afr. Health Sci..

[122] Bahijri S., Borai A., Ajabnoor G., Abdul Khaliq A., AlQassas I., Al-Shehri D., Chrousos G. (2013). Relative metabolic stability, but disrupted circadian cortisol secretion during the fasting month of Ramadan. PLoS ONE.

[123] Ajabnoor G.M., Bahijri S., Shaik N.A., Borai A., Alamoudi A.A., Al-Aama J.Y., Chrousos G.P. (2017). Ramadan fasting in Saudi Arabia is associated with altered expression of CLOCK, DUSP and IL-1alpha genes, as well as changes in cardiometabolic risk factors. PLoS ONE.

[124] Al-Hadramy M.S., Zawawi T.H., Abdelwahab S.M. (1988). Altered cortisol levels in relation to Ramadan. Eur. J. Clin. Nutr..

[125] Riat A., Suwandi A., Ghashang S.K., Buettner M., Eljurnazi L., Grassl G.A., Gutenbrunner C., Nugraha B. (2021). Ramadan Fasting in Germany (17–18 h/Day): Effect on Cortisol and Brain-Derived Neurotrophic Factor in Association With Mood and Body Composition Parameters. Front. Nutr..

[126] Marciniak M., Sato M., Rutkowski R., Zawada A., Juchacz A., Mahadea D., Grzymislawski M., Dobrowolska A., Kawka E., Korybalska K. (2023). Effect of the one-day fasting on cortisol and DHEA daily rhythm regarding sex, chronotype, and age among obese adults. Front. Nutr..

[127] Fichter M.M., Pirke K.M., Holsboer F. (1986). Weight loss causes neuroendocrine disturbances: Experimental study in healthy starving subjects. Psychiatry Res..

[128] Shibuya I., Nagamitsu S., Okamura H., Komatsu H., Ozono S., Yamashita Y., Matsuishi T. (2011). Changes in salivary cortisol levels as a prognostic predictor in children with anorexia nervosa. Int. J. Psychophysiol..

[129] Vanderschueren S., Geens E., Knockaert D., Bobbaers H. (2005). The diagnostic spectrum of unintentional weight loss. Eur. J. Intern. Med..

[130] Whitworth J.A., Williamson P.M., Mangos G., Kelly J.J. (2005). Cardiovascular consequences of cortisol excess. Vasc. Health Risk Manag..

[131] Heim C., Ehlert U., Hellhammer D.H. (2000). The potential role of hypocortisolism in the pathophysiology of stress-related bodily disorders. Psychoneuroendocrinology.

[132] Hannibal K.E., Bishop M.D. (2014). Chronic stress, cortisol dysfunction, and pain: A psychoneuroendocrine rationale for stress management in pain rehabilitation. Phys. Ther..

[133] Invitti C., Redaelli G., Baldi G., Cavagnini F. (1999). Glucocorticoid receptors in anorexia nervosa and Cushing’s disease. Biol. Psychiatry.

[134] Kuo T., McQueen A., Chen T.C., Wang J.C. (2015). Regulation of Glucose Homeostasis by Glucocorticoids. Adv. Exp. Med. Biol..

[135] Laycock J., Meeran K. (2012). Integrated Endocrinology.

[136] Licinio C., Mantzoros A.B., NegraÄo G., Cizza M.L., Wong P.B., Bongiorno G.P., Chrousos B., Karp C., Allen J.S., Flier P.W. (1997). Human leptin levels are pulsatile and inversely related to pituitary-adrenal function. Nat. Med..

[137] Flier J.S. (1998). Clinical review 94. What’s in a name? In search of leptin’s physiologic role. J. Clin. Endocrinol. Metab..

[138] Prolo P., Wong M., Licinio J. (1998). Leptin. Int. J. Biochem. Cell Biol..

[139] Malczyk Z., Roczniak W., Mazur B., Kwiecien J., Ziora K., Górska-Flak K., Oswiecimska J. (2021). Exocrine Pancreatic Function in Girls with Anorexia Nervosa. Nutrients.

[140] Campbell J.E., Drucker D.J. (2015). Islet alpha cells and glucagon-critical regulators of energy homeostasis. Nat. Rev. Endocrinol..

[141] Zhang Z.N., Gong L., Lv S., Li J., Tai X., Cao W., Peng B., Qu S., Li W., Zhang C. (2016). SIK2 regulates fasting-induced PPARα activity and ketogenesis through p300. Sci. Rep..

[142] Rah S.Y., Joe Y., Park J., Ryter S.W., Park C., Chung H.T., Kim U.H. (2023). CD38/ADP-ribose/TRPM2-mediated nuclear Ca^2+^ signaling is essential for hepatic gluconeogenesis in fasting and diabetes. Exp. Mol. Med..

[143] Jiang G., Zhang B.B. (2003). Glucagon and regulation of glucose metabolism. Am. J. Physiol. Endocrinol. Metab..

[144] Galsgaard K.D., Pedersen J., Knop F.K., Holst J.J., Wewer Albrechtsen N.J. (2019). Glucagon Receptor Signaling and Lipid Metabolism. Front. Physiol..

[145] Wahren J., Ekberg K. (2007). Splanchnic regulation of glucose production. Annu. Rev. Nutr..

[146] Rothman D.L., Magnusson I., Katz L.D., Shulman R.G., Shulman G.I. (1991). Quantitation of hepatic glycogenolysis and gluconeogenesis in fasting humans with 13C NMR. Science.

[147] Schauder P., Herbertz L., Langenbeck U. (1985). Serum branched chain amino and keto acid response to fasting in humans. Metabolism.

[148] Yamamoto K., Omura D., Yamane M., Son R., Hasegawa K., Honda H., Obika M., Minao N., Edahiro S., Yamada N. (2021). Recurrence of Hypoglycemic Coma in a Patient with Anorexia Nervosa. Acta Med. Okayama.

[149] Anyetei-Anum C.S., Roggero V.R., Allison L.A. (2018). Thyroid hormone receptor localization in target tissues. J. Endocrinol..

[150] Galasko G.T. (2017). Pituitary, Thyroid, and Parathyroid Pharmacology. Pharmacology and Therapeutics for Dentistry.

[151] Sinha R., Yen P.M., Feingold K.R., Anawalt B., Blackman M.R., Boyce A., Chrousos G., Corpas E., de Herder W.W., Dhatariya K., Dungan K., Hofland J. (2000). Cellular Action of Thyroid Hormone. Endotext [Internet].

[152] Brent G.A. (2012). Mechanisms of thyroid hormone action. J. Clin. Investig..

[153] Eom Y.S., Wilson J.R., Bernet V.J. (2022). Links between Thyroid Disorders and Glucose Homeostasis. Diabetes Metab. J..

[154] Fedail S.S., Murphy D., Salih S.Y., Bolton C., Harvey R.F. (1982). Changes in certain blood constituents during Ramadan. Am. J. Clin. Nutr..

[155] Basolo A., Begaye B., Hollstein T., Vinales K.L., Walter M., Santini F., Krakoff J., Piaggi P. (2019). Effects of Short-Term Fasting and Different Overfeeding Diets on Thyroid Hormones in Healthy Humans. Thyroid.

[156] Samuels M., Kramer P. (1996). Differential effects of short-term fasting on pulsatile thyrotropin, gonadotropin, and alpha-subunit secretion in healthy men—A clinical research center study. J. Clin. Endocrinol. Metab..

[157] Chait A., den Hartigh L.J. (2020). Adipose Tissue Distribution, Inflammation and Its Metabolic Consequences, Including Diabetes and Cardiovascular Disease. Front. Cardiovasc. Med..

[158] Tabuchi C., Sul H.S. (2021). Signaling Pathways Regulating Thermogenesis. Front. Endocrinol..

[159] Yau W.W., Yen P.M. (2020). Thermogenesis in Adipose Tissue Activated by Thyroid Hormone. Int. J. Mol. Sci..

[160] Ucci S., Renzini A., Russi V., Mangialardo C., Cammarata I., Cavioli G., Santaguida M.G., Virili C., Centanni M., Adamo S. (2019). Thyroid Hormone Protects from Fasting-Induced Skeletal Muscle Atrophy by Promoting Metabolic Adaptation. Int. J. Mol. Sci..

[161] Tomova A., Kumanov P. (1999). Sex differences and similarities of hormonal alterations in patients with anorexia nervosa. Andrologia.

[162] Kim B.H., Joo Y., Kim M.S., Choe H.K., Tong Q., Kwon O. (2021). Effects of Intermittent Fasting on the Circulating Levels and Circadian Rhythms of Hormones. Endocrinol. Metab..

[163] Agnihothri R.V., Courville A.B., Linderman J.D., Smith S., Brychta R., Remaley A., Chen K.Y., Simchowitz L., Celi F.S. (2014). Moderate weight loss is sufficient to affect thyroid hormone homeostasis and inhibit its peripheral conversion. Thyroid.

[164] Jada K., Djossi S.K., Khedr A., Neupane B., Proskuriakova E., Mostafa J.A. (2021). The Pathophysiology of Anorexia Nervosa in Hypothalamic Endocrine Function and Bone Metabolism. Cureus.

[165] Azizi F. (2015). Islamic fasting and thyroid hormones. Intl J. Endocrinol. Metab..

[166] Flier J.S., Maratos-Flier E. (2017). Leptin’s Physiologic Role: Does the Emperor of Energy Balance Have No Clothes?. Cell Metab..

[167] Flier J.S., Harris M., Hollenberg A.N. (2000). Leptin, nutrition, and the thyroid; the why, the wherefore, and the wiring. J. Clin. Investig..

[168] Herpertz S., Wagner R., Albers N., Blum W.F., Pelz B., Langkafel M., Köpp W., Henning A., Oberste-Berghaus C., Mann K. (1998). Circadian plasma leptin levels in patients with anorexia nervosa: Relation to insulin and cortisol. Horm. Res..

[169] Park H.K., Ahima R. (2015). Physiology of leptin: Energy homeostasis, neuroendocrine function and metabolism. Metabolism.

[170] Himms-Hagen J. (1990). Brown adipose tissue thermogenesis: Interdisciplinary studies. FASEB J..

[171] Chan J.L., Mantzoros C.S. (2005). Role of leptin in energy-deprivation states: Normal human physiology and clinical implications for hypothalamic amenorrhoea and anorexia nervosa. Lancet.

[172] Rupert J.E., Jengelley D.H.A., Zimmers T.A. (2020). In Vitro, In Vivo, and In Silico Methods for Assessment of Muscle Size and Muscle Growth Regulation. Shock.

[173] Chandramouli V., Ekberg K., Schumann W.C., Kalhan S.C., Wahren J., Landau B.R. (1997). Quantifying gluconeogenesis during fasting. Am. J. Physiol..

[174] Lithell H., Boberg J., Hellsing K., Lundqvist G., Vessby G. (1978). Lipoproteinlipase activity in human skeletal muscle and adipose tissue in the fasting and the fed states. Atherosclerosis.

[175] Samra J.S., Clark M.L., Humphreys S.M., Macdonald I.A., Frayn K.N. (1996). Regulation of lipid metabolism in adipose tissue during early starvation. Am. J. Physiol..

[176] Lass A., Zimmermann R., Oberer M., Zechner R. (2011). Lipolysis—A highly regulated multi-enzyme complex mediates the catabolism of cellular fat stores. Prog. Lipid Res..

[177] Djurhuus C.B., Gravholt C.H., Nielsen S., Mengel A., Christiansen J.S., Schmitz O.E., Møller N. (2002). Effects of cortisol on lipolysis and regional interstitial glycerol levels in humans. Am. J. Physiol. Endocrinol. Metab..

[178] Fong H.F., Divasta A.D., Difabio D., Ringelheim J., Jonas M.M., Gordon C.M. (2008). Prevalence and predictors of abnormal liver enzymes in young women with anorexia nervosa. J. Pediatr..

[179] Walsh B.T., Katz J.L., Levin J., Kream J., Fukushima D.K., Hellman L.D., Weiner H., Zumoff B. (1978). Adrenal activity in anorexia nervosa. Psychosom. Med..

[180] Peters A., Rohloff D., Kohlmann T., Renner F., Jantschek G., Kerner W., Fehm H.L. (1998). Fetal hemoglobin in starvation ketosis of young women. Blood.

[181] Salti I., Bénard E., Detournay B., Bianchi-Biscay M., Le Brigand C., Voinet C., Jabbar A. (2004). Results of the Epidemiology of Diabetes and Ramadan 1422/2001 (EPIDIAR) study. Diabetes Care.

[182] Velayudhan M. (2012). Managing diabetes during the Muslim fasting month of Ramadan. Med. J. Malaysia..

[183] Bakiner O., Ertorer M.E., Bozkirli E., Tutuncu N.B., Demirag N.G. (2009). Repaglinide plus single-dose insulin glargine: A safe regimen for low-risk type 2 diabetic patients who insist on fasting in Ramadan. Acta Diabetol..

[184] Toni G., Berioli M.G., Cerquiglini L., Ceccarini G., Grohmann U., Principi N., Esposito S. (2017). Eating Disorders and Disordered Eating Symptoms in Adolescents with Type 1 Diabetes. Nutrients.

[185] Ji J., Sundquist J., Sundquist K. (2016). Association between anorexia nervosa and type 2 diabetes in Sweden: Etiological clue for the primary prevention of type 2 diabetes. Endocr. Res..

[186] Carneiro L., Geller S., Hébert A., Repond C., Fioramonti X., Leloup C., Pellerin L. (2016). Hypothalamic sensing of ketone bodies after prolonged cerebral exposure leads to metabolic control dysregulation. Sci. Rep..

[187] Haines M.S. (2023). Endocrine complications of anorexia nervosa. J. Eat. Disord..

[188] Yu Y., Huang R., Ye J., Zhang V., Wu C., Cheng G., Jia J., Wang L. (2016). Regulation of starvation-induced hyperactivity by insulin and glucagon signaling in adult Drosophila. eLife.

[189] Hatting M., Tavares C.D., Sharabi K., Rines A.K., Puigserver P. (2018). Insulin regulation of gluconeogenesis. Ann. N. Y. Acad. Sci..

[190] Rodwell V.W., Bender D.A., Botham K.M., Kennelly P.J., Weil P.A. (2018). Harper’s Illustrated Biochemistry.

[191] Stratton M.T., Albracht-Schulte K., Harty P.S., Siedler M.R., Rodriguez C., Tinsley G.M. (2022). Physiological responses to acute fasting: Implications for intermittent fasting programs. Nutr. Rev..

[192] Ohwada R., Hotta M., Oikawa S., Takano K. (2006). Etiology of hypercholesterolemia in patients with anorexia nervosa. Int. J. Eat. Disord..

[193] Kraus-Friedmann N. (1984). Hormonal regulation of hepatic gluconeogenesis. Physiol. Rev..

[194] Spaulding S.W., Chopra I.J., Sherwin R.S., Lyall S.S. (1976). Effect of caloric restriction and dietary composition of serum T3 and reverse T3 in man. J. Clin. Endocrinol. Metab..

[195] Schebendach J.E., Golden N.H., Jacobson M.S., Hertz S., Shenker I.R. (1997). The metabolic responses to starvation and refeeding in adolescents with anorexia nervosa. Ann. N. Y. Acad. Sci..

[196] Hübel C., Yilmaz Z., Schaumberg K.E., Breithaupt L., Hunjan A., Horne E., García-González J., O’Reilly P.F., Bulik C.M., Breen G. (2019). Body composition in anorexia nervosa: Meta-analysis and meta-regression of cross-sectional and longitudinal studies. Int. J. Eat. Disord..

[197] Misra M., Miller K.K., Kuo K., Griffin K., Stewart V., Hunter E., Herzog D.B., Klibanski A. (2005). Secretory dynamics of leptin in adolescent girls with anorexia nervosa and healthy adolescents. Am. J. Physiol. Endocrinol. Metab..

[198] Bianco A.C., Maia A.L., da Silva W.S., Christoffolete M.A. (2005). Adaptive activation of thyroid hormone and energy expenditure. Biosci. Rep..

[199] Araujo R.L., de Andrade B.M., de Figueiredo Á.S.P., da Silva M.L., Marassi M.P., dos Santos V., Bouskela E., Carvalho D.P. (2008). Low replacement doses of thyroxine during food restriction restores type 1 deiodinase activity in rats and promotes body protein loss. J. Endocrinol..

[200] Schweizer U., Steegborn C. (2015). New insights into the structure and mechanism of iodothyronine deiodinases. J. Mol. Endocrinol..

[201] da Silveira C.D., de Vasconcelos F.P., Moura E.B., da Silveira B.T., Amorim F.F., Shintaku L.S., de Santana R.B., Argotte P.L., da Silva S.F., de Oliveira M. (2021). Thyroid Function, Reverse Triiodothyronine, and Mortality in Critically Ill Clinical Patients. Indian. J. Crit. Care Med..

[202] Elliott B., Mina M., Ferrier C. (2016). Complete and Voluntary Starvation of 50 days. Clin. Med. Insights Case Rep..

[203] BBC The Full Story of Thailand’s Extraordinary Cave Rescue. https://www.bbc.com/news/world-asia-44791998.

[204] Bailer U.F., Kaye W.H. (2003). A review of neuropeptide and neuroendocrine dysregulation in anorexia and bulimia nervosa. Curr. Drug Targets CNS Neurol. Disord..

[205] Randle P.J. (1995). Metabolic fuel selection: General integration at the whole body level. Proc. Nutr. Soc..

[206] Peters A. (2011). The selfish brain: Competition for energy resources. Am. J. Hum. Biol..

