# Supplementary material for: Adaptive Effects of Endocrine Hormones on Metabolism of Macronutrients during Fasting and Starvation: A Scoping Review
Source: Metabolites. 2024 Jun 16;14(6):336. doi: 10.3390/metabo14060336 (PMC11205672; doi:10.3390/metabo14060336)
Supplement: Supplementary file 1 [file metabolites-14-00336-s001.zip › metabolites-2965355-supplementary.pdf]

**Table S1.** Identified scientific literature resources containing pertinent information on adaptive effects of endocrine hormones on human metabolism of endogenous macronutrients during fasting and starvation

| **Endocrine organ (Hormone)** | **First author, year of publication, and [reference]** | **Type of article (Country of research)** | **Human population (Gender)** | **Fasting or Starvation** | **Key findings that relate to the study question** |
|---|---|---|---|---|---|
| **Adrenal glands (cortisol)** | Koutkia et al., 2003, [32] | Clinical study, (USA) | 20 (Males) | Fasting | ↑Cortisol |
| | Gamble et al., 2014, [43] | Review, (USA) | NA | | ↑Cortisol |
| | Yu et al., 2011, [45] | Clinical study, (China) | 19 (9 Males and 10 Females) | | ↑Cortisol |
| | Dimitriadis, et al., 2021, [47] | Review, (United Kingdom) | NA | | ↑Glycogenolysis<br>↓Peripheral tissue glucose uptake and utilization |
| | Oh, et al., (2013), [48] | Review, (South Korea) | NA | | ↑Glycogenolysis |
| | *Magyar (2022), [50]* | *Clinical study, (USA)* | *20 (Females)* | | ↓*Cortisol* |
| | Casanueva, & Dieguez, (1999), [49] | Review, (Spain) | NA | | ↓Leptin leading to ↑Cortisol |
| | Bergendahl et al., 2000, [44] | Clinical study, (Finland) | 6 (Females) | | ↑Cortisol |
| | Oh, et al., (2013), [46] | Review, (South Korea) | NA | | ↑Gluconeogenesis |
| | Chan et al., 2003, [29] | Clinical study, (USA) | 8 (Males) | | ↑Cortisol |
| | Douyon & Schteingart, 2002 [20] | Review, (USA) | NA | Starvation | ↑Cortisol |
| | Amorim et al., 2023, [36] | Review, (USA) | NA | | ↑Cortisol |
| | Paszynska, et al., (2016), [51] | Clinical study, (Poland) | 47 (Females) | | ↑Cortisol |

| | | | | | |
|---|---|---|---|---|---|
| | Herpertz et al., (2000), [52] | Clinical study, (Germany) | 5 (1 Male and 4 Females) | | ↑Cortisol |
| | Wassif & Ross, (2013), [53] | Book Chapter, (United Kingdom) | NA | | ↑Cortisol |
| | Thavaraputta et al., 2023, [54] | Review, (USA) | NA | | ↑Cortisol |
| | Boyar et al., (1977), [55] | Clinical study, (USA) | 10 (Females) | | ↑Cortisol |
| | Takahara et al., (1976), 56] | Clinical study, (Japan) | 14 (1 Male and 13 Females) | | ↑Cortisol |
| | Gwirtsman, et al., (1989), [57] | Clinical study, (USA) | 16 (Females) | | ↑Cortisol |
| | Casper et al., (1979), [58] | Clinical study, (USA) | 20 (Females) | | ↑Cortisol |
| | Elegido et al., (2019), [59] | Clinical study, (Spain) | 41 (Females) | | ↑Cortisol |
| | Gold et al., (1986), [60] | Clinical study, (USA) | 15 (Female) | | ↑Cortisol |
| | Salisbury & Mitchell, (1991), [61] | Review, (USA) | NA | | ↑Cortisol |
| | Misra & Klibanski, (2010), [62] | Review, (USA) | NA | | ↑Gluconeogenesis |
| | Moyano et al., 1998, [63] | Clinical* study, (Spain) | 92 (Females) | | ↑Amino acids |
| **Pancreas (glucagon & insulin)** | Goldstein & Hager, (2018), [64] | Review, (USA) | NA | Fasting | ↑Glucagon |
| | Andersen & Holst (2021), [65] | Review, (Denmark) | NA | | ↑Glucagon |
| | Saltiel, (2016), [66] | Review, (USA) | NA | | ↑Glucagon |
| | Massa, et al., (2011), [69] | Review, (Argentina) | NA | | ↑Glucagon |
| | Nakata, & Yada (2007), [67] | Review, (Japan) | NA | | ↑Glucagon |

| | | | | | |
|---|---|---|---|---|---|
| | Zhang et al., (2019), [68] | Review, (China) | NA | | ↑Glucagon |
| | Vardarli, et al., (2014), [37] | Clinical study, (Germany) | 19 (Males) | | Fasting's benefits |
| | van den Berghe, (1991), [70] | Review, (USA) | NA | | ↑Glycogenolysis |
| | Taborsky, (2010), [71] | Review, (USA) | NA | | ↑Glycogenolysis |
| | Sharabi et al., (2019), [72] | Review, (USA) | NA | | ↑Glycogenolysis |
| | Klover & Mooney, (2003), [35] | Review, (USA) | NA | | ↑Glycogenolysis<br>↓Glycogenesis |
| | Docherty & Clark, (1994), [73] | Review, (United Kingdom) | NA | | ↓Insulin |
| | Habegger (2022), [74] | Review, (USA) | NA | | ↓Insulin |
| | Ahima (2006), [75] | Review, (USA) | NA | | ↑Glucagon<br>↓Insulin<br>↑Glycogenolysis<br>↑Lipolysis<br>↑Ketogenesis |
| | Bröer & Bröer, (2017), [77] | Review, (USA) | NA | | ↑Gluconeogenesis<br>↑Amino acids oxidation<br>↑Ureagenesis |
| | Popovic & Duntas, (2005), [34] | Review (Serbia) | NA | | ↑Cortisol<br>↓Insulin<br>↓Leptin |
| | Ahima et al., (2006), [78] | Review, (USA) | NA | | ↑Cortisol<br>↓Insulin<br>↓Leptin<br>↓$T_3$ |
| | Kamagate & Dong, (2008), [76] | Review, (USA) | NA | | ↓Insulin<br>↑Gluconeogenesis |
| | Daval et al., (2006), [79] | Review, (France) | NA | | ↑Lipolysis |
| | Carlson et al., 1994, [80] | Clinical study, (USA) | 6 (Males) | | ↑Proteolysis<br>↑Lipolysis |
| | Heruc et al., (2018), [81] | Clinical study, (Australia) | 22 (Females) | Starvation | ↑Glucagon |

| | | | | | |
|---|---|---|---|---|---|
| | Blickle, et al., (1984), [15] | Clinical study, (Germany) | 26 (Females) | | ↑Glucagon |
| | Kumai et al., (1988), [82] | Clinical study, (Japan) | 25 (Females) | | ↑Glucagon |
| | Alderdice et al., (1985), [92] | *Clinical study, (Ireland)* | *8 (Females)* | | ↓*Glucagon* |
| | Casper, 1996, [83] | Review, (USA) | NA | | ↑Lipolysis ↑Ketogenesis |
| | Weinbrenne, et al., (2004), [84] | Clinical study, (Germany) | 58 (Females) | | ↓Insulin |
| | Misra et al., (2004), [85] | Clinical study, (USA) | 23 (Females) | | ↓Insulin |
| | Mocanu et al., 2003 [86] | Clinical study, (France) | 142 (Females) | | ↓Insulin |
| | Franssila-Kallunki et al., (1991), [87] | Clinical study, (Finland) | 11 (Females) | | ↓Insulin |
| | Tural & Iosifescu, (2022), [88] | Review, (USA) | NA | | ↓Insulin |
| | Dostálová et al., (2007), [89] | Clinical study, (Czech Republic) | 10 (Females) | | ↓Insulin |
| | Fonseca, et al., (1991), [19] | Clinical study, (United Kingdom) | 1 (Female) | | ↓Insulin |
| | Ilyas et al., (2019), [93] | *Review, (United Kingdom)* | *NA* | | ↑*Insulin sensitivity* |
| | Ho et al., (1988), [90] | Clinical study, (USA) | 6 (Males) | | ↑Fatty acid oxidation ↑Ketogenesis |
| | Misra & Klibanski, 2014, [16] | Review, (USA) | NA | | ↑Lipolysis ↑Gluconeogenesis |
| | Fehm et al., (2006), [91] | Review, (Germany) | NA | | ↑Competition for energy resources |

| | | | | | |
|---|---|---|---|---|---|
| | Martinez & Ortiz, 2017, [38] | Review, (USA) | NA | Fasting and Starvation | ↑Glucagon<br>↓Insulin<br>↓$T_3$ |
| **Thyroid gland (thyroid hormones)** | van der Spek et al., (2017) [94] | Review, (Netherlands ) | NA | Fasting | ↓$T_3$<br>↓TSH<br>↓TRH |
| | Iwen et al., (2018), [95] | Review, (Germany) | NA | | ↓$T_3$<br>↓TSH<br>↓TRH |
| | Boelen, et al., (2008), [96] | Review, (Netherlands ) | NA | | ↓TSH |
| | Lechan & Fekete, (2006), [97] | Review, (USA) | NA | | ↓TRH |
| | Fekete & Lechan, (2007), [98] | Review, (Hungary) | NA | | ↓$T_3$<br>↓TRH |
| | Das et al., (2018), [39] | Review, (Canada) | NA | | Fasting's benefits |
| | Janeckova (2001), [99] | Review (Czech Republic) | NA | | ↓Leptin leading to ↓$T_3$ |
| | de Rosa ET AL., (1983), [18] | Clinical study, (Italy) | 23 (3 Males and 20 Females) | Starvation | ↑r$T_3$<br>↓$T_3$ |
| | Curran-Celentano et al., (1985), [21] | Clinical study, (USA) | 14 (1 Male and 20 Females) | | ↑r$T_3$<br>↓$T_3$ |
| | Moore & Mills, (1979), [25] | Clinical study, (England) | 33 (Females) | | ↓$T_3$ |
| | Støving et al., (1999), [100] | Review, (Denmark) | NA | | ↓$T_3$ |
| | Schreiber et al., (1991), [17] | Clinical study, (Germany) | 10 (Females) | | ↓$T_3$ |
| | Komaki et al., (1992), [22] | Clinical study, (Japan) | 9 (1 Male and 8 Females) | | ↓$T_3$ |
| | Bannai et al., (1988), [23] | Clinical study, (Japan) | 16 (Females) | | ↓$T_3$ |
| | Leslie et al., (1978), [24] | Clinical study, (England) | 14 (Females) | | ↓$T_3$ |

| | | | | | |
|---|---|---|---|---|---|
| | Moshang et al., (1975), [27] | Clinical study, (USA) | 6 (Females) | | ↓$T_3$ |
| | de Rosa et al., 1983, [28] | Clinical study, (Italy) | 20 (Females) | | ↓$T_3$ |
| | Schorr & Miller, (2017), [105] | Review, (USA) | NA | | ↓$T_3$<br>↑$rT_3$ |
| | Croxson & Ibbertson, (1977), [101] | Clinical study, (New Zealand) | 22 (2 Males and 20 Females) | | ↓$T_3$ |
| | Capo-chichi et al., (1999), [102] | Clinical study, (France) | 17 (Females) | | ↓$T_3$ |
| | Onur et al., (2005), [103] | Clinical study, (Germany) | 28 (Females) | | ↓$T_3$ |
| | Miyai et al., (1975), [104] | Clinical study, (Japan) | 16 (Females) | | ↓$T_3$ |
| | Tamai et al., 1986, [26] | Clinical study, (Japan) | 21 (Females) | | ↓TSH<br>↓TBG |
| | Reinehr et al., (2008), [33] | Clinical study, (Germany) | 20 (Females) | | ↓$T_3$<br>↓TSH |
| | Kiyohara et al., (1989), [106] | Clinical study, (Japan) | 10 (Females) | | ↓$T_3$<br>↓TSH |
| | Pannacciulli et al., (2003), [107] | Clinical study, (Italy) | 11 (Females) | | ↓Leptin |

*While no direct measurement of cortisol was undertaken, authors claim cortisol might support the metabolic basis for high plasma amino acids in patients with AN. Text in italics represents contradicting results.

Upward arrow (↑) indicates "increase", downward arrow (↓) indicates "reduction", for a hormone level or metabolic reaction during the indicated food deprivation.

NA: Not Applicable; TRH: thyrotropin-releasing hormone; $T_3$: triiodothyronine; $rT_3$: reverse $T_3$; TSH: thyroid-stimulating hormone; TBG: thyroxine-binding globulin
